# The intra-mitochondrial O-GlcNAcylation system rapidly modulates OXPHOS function and ROS release in the heart

**DOI:** 10.1038/s42003-022-03282-3

**Published:** 2022-04-12

**Authors:** Justine Dontaine, Asma Bouali, Frederic Daussin, Laurent Bultot, Didier Vertommen, Manon Martin, Raahulan Rathagirishnan, Alexanne Cuillerier, Sandrine Horman, Christophe Beauloye, Laurent Gatto, Benjamin Lauzier, Luc Bertrand, Yan Burelle

**Affiliations:** 1 Pole of Cardiovascular Research (CARD), Institute of Experimental and Clinical Research (IREC), UCLouvain, Brussels, Belgium; 2 Interdisciplinary School of Health Sciences, Faculty of Health Sciences, University of Ottawa, Ottawa, ON Canada; 3 Univ. Lille, Univ. Artois, Univ. Littoral Côte d’Opale, ULR 7369 - URePSSS - Unité de Recherche Pluridisciplinaire Sport Santé Société, F-59000 Lille, France; 4 Pole of Protein phosphorylation (PHOS) and proteomic platform (MASSPROT), de Duve Institute (DDUV), UCLouvain, Brussels, Belgium; 5 Pole of Computational biology and bioinformatics (CBIO), de Duve Institute (DDUV), UCLouvain, Brussels, Belgium; 6 Department of Cellular and Molecular Medicine, Faculty of Medicine, University of Ottawa, Ottawa, ON Canada; 7 Division of Cardiology, Cliniques Universitaires Saint-Luc, Université Catholique de Louvain, Brussels, Belgium; 8 Institute of Thorax, INSERM, CNRS, University of Nantes, Nantes, France; 9 WELBIO, Walloon Excellence in Life Sciences and BIOtechnology, Brussels, Belgium

**Keywords:** Energy metabolism, Post-translational modifications

## Abstract

Protein O-GlcNAcylation is increasingly recognized as an important cellular regulatory mechanism, in multiple organs including the heart. However, the mechanisms leading to O-GlcNAcylation in mitochondria and the consequences on their function remain poorly understood. In this study, we use an in vitro reconstitution assay to characterize the intra-mitochondrial O-GlcNAc system without potential cytoplasmic confounding effects. We compare the O-GlcNAcylome of isolated cardiac mitochondria with that of mitochondria acutely exposed to NButGT, a specific inhibitor of glycoside hydrolase. Amongst the 409 O-GlcNAcylated mitochondrial proteins identified, 191 display increased O-GlcNAcylation in response to NButGT. This is associated with enhanced Complex I (CI) activity, increased maximal respiration in presence of pyruvate-malate, and a striking reduction of mitochondrial ROS release, which could be related to O-GlcNAcylation of specific subunits of ETC complexes (CI, CIII) and TCA cycle enzymes. In conclusion, our work underlines the existence of a dynamic mitochondrial O-GlcNAcylation system capable of rapidly modifying mitochondrial function.

## Abstract (web summary)

An in vitro assay in isolated heart mitochondria reveals that O-GlcNAcase inhibitor NButGT rapidly increases protein O-GlcNAcylation leading to increased respiratory capacity and complex I activity and decreased ROS release.

## Introduction

O-linked N-acetylglucosamination (O-GlcNAcylation) is a dynamic post-translational modification of proteins characterized by the addition of a single acetylated hexosamine moiety to certain Ser/Thr residues through O-linkage on their hydroxyl^1,2^. Protein O-GlcNAcylation requires uridine disphosphate N-acetylglucosamine (UDP-GlcNAc) as substrate, which is mainly derived from glucose through the hexosamine biosynthesis pathway (HBP), driven by its rate limiting enzyme the glutamine:fructose-6-phosphate amidotransferase (GFAT). O-GlcNAcylation is regulated by the uridine diphospho-N-acetylglucosamine transferase (OGT), and the O-GlcNAcase (OGA), which respectively add and remove O-GlcNAc moieties^1,2^. These highly conserved and ubiquitously expressed enzymes are present in various cellular locations where they regulate several protein properties such as their activation state, localization, stability and/or degradation^3^. Over the past several years, protein O-GlcNAcylation has emerged as both pathogenic factor^4^ and important mechanism involved in physiological processes such as development and protection against cellular stress^5,6^. Such dual action can be found in the heart. Indeed, chronic elevation of protein O-GlcNAcylation is believed to participate in the development of metabolic and contractile dysfunctions associated with diabetes^7^ and cardiac hypertrophy^2,8^. Conversely, acute hyper O-GlcNAcylation is known to confer protection against ischaemic damage^6^ and sepsis-induced contractile dysfunction^9,10^, which has contributed to position protein O-GlcNAcylation as a potential therapeutic target for the management of both chronic and acute cardiovascular conditions.

While OGT and OGA are mainly localized to the nuclear and cytosolic compartments, several studies have shown that mitochondria are major targets for O-GlcNAcylation^1,11^. Furthermore, growing evidence suggest that mitochondrial protein O-GlcNAcylation in fact plays a role not only in the development of diabetic cardiomyopathy^12,13^, but also in the cardio-protective effect of acute hyper O-GlcNAcylation^6,14^. This notion has been recently reinforced by results showing the presence of an UDP-GlcNAc carrier along with OGA and a 103 kDa isoform of OGT (mOGT) in the mitochondrial compartment^13,15,16^. Some controversies nevertheless exist regarding the presence of these enzymes in the mitochondrial compartment^17^. More importantly, since mitochondrial O-GlcNAcylation has mainly been investigated in cell culture models or in vivo, the direct effects of the putative mitochondrial O-GlcNAc cycling system have been difficult to distinguish from the indirect effects mediated by the nucleocytoplasmic O-GlcNAcylation system.

In this study, we therefore took advantage of an in vitro reconstitution assay to characterize the intra-mitochondrial O-GlcNAcylation system in isolated cardiac mitochondria. Our results confirm the presence of a fully functional and dynamic O-GlcNAc cycling system in these organelles. Using comparative O-GlcNAc proteomics (O-GlcNAcylomics), we provide evidence that the local mitochondrial O-GlcNAcylation system can trigger broad and rapid changes in protein O-GlcNAcylation, which are highly reminiscent of the mitochondrial O-GlcNAcylation profile observed in vivo. Importantly, we also reveal that acute hyper-O-GlcNAcylation increases maximal respiratory capacity, and drastically reduces ROS release though a complex-I mediated mechanism, illustrating the capacity of this system to rapidly modify mitochondrial function.

## Results

### In vitro reconstitution assay allows to target the intra-mitochondrial O-GlcNAcylation system

In order to characterize the mitochondrial O-GlcNAcylation system without the potential confounding effects of the O-GlcNAcylation in other cellular compartments, we devised an in vitro reconstitution assay in which isolated cardiac mitochondria were acutely exposed (*i.e*. 30 min) to the OGT substrate UDP-GlcNAc in presence or absence of the OGA inhibitor NButGT, with the goal of inducing rapid changes in protein O-GlcNAcylation levels.

**Fig. 1:**
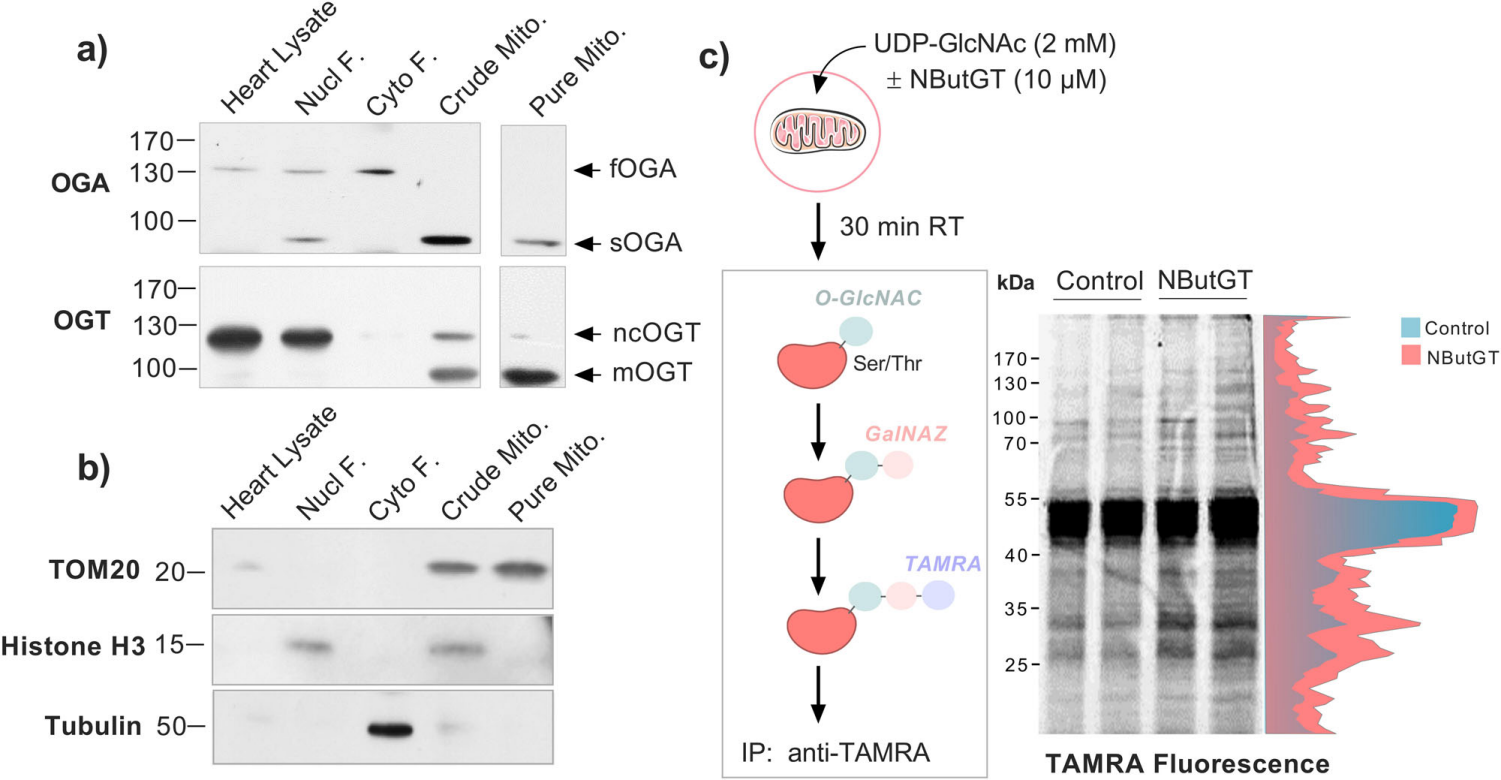
Characterization of the in vitro reconstitution assay used to investigate the mitochondrial O-GlcNAcylation cycling system. **a** Representative western-blot of OGA and OGT protein content measured in the whole heart homogenate, nuclear, cytosolic, crude, and Percoll-purified mitochondrial fractions (Pure Mito). Note that the image shown for the pure mitochondrial fraction is derived from a separate blot. **b** Representative western-blot of the relative expression of mitochondrial (TOM20), nuclear (Histone H3), and cytosolic (Tubulin) marker proteins in the various cellular fractions. **c** Overview of the in vitro reconstitution assay used to induce and detect mitochondrial protein O-GlcNAcylation in isolated mitochondria. Crude cardiac mitochondrial fractions isolated from rat hearts were incubated at room temperature in presence of UDP-GlcNAc in the absence of presence of the OGA inhibitor NButGT. After 30 min, proteins were denatured and incubated with UDP-GalNAz in presence of the enzyme Y289L GalT to label O-GlcNAc-modified proteins. The GalNAz adducts obtained were functionalized with TAMRA, and an anti-TAMRA antibody was subsequently used to immunocapture O-GlcNAc-modified proteins. Immunoprecipitates enriched with O-GlcNAc-modified proteins were resolved by electrophoresis and revealed by fluorescence at the wavelength of TAMRA (570 nm). Following densitometric quantification of band intensity (shown in red and blue), gels were cut in 7 pieces of equal size and further processed for proteomics analysis.

As some controversy exists regarding the expression of the mOGT isoform in murine tissues^17^, and the presence of sufficient OGA in the mitochondrial compartment^13^, the presence of these enzymes was first verified by immunoblotting in whole lysates from crude and Percoll-purified mitochondria (Fig. 1a). Specific protein markers were firstly assessed to confirm the purity of the different purified fractions (Fig. 1b). As expected, the mitochondrial marker TOM20 was highly enriched in the crude and Percoll-purified mitochondrial fractions, while histone H3 and alpha tubulin were mostly recovered in the nuclear and cytosolic fractions, respectively. Small amounts of histone H3 and alpha-tubulin remained present in the crude mitochondrial preparation, but were largely removed by the Percoll purification step. As represented in the Fig. 1a, immunoblotting of crude mitochondrial fractions with anti-OGT antibody revealed a predominant band at 103 kDa, which corresponds to the expected molecular weight of the mitochondrial isoform mOGT^15^. A band was also observed at 116 kDa, consistent with the presence of the nucleocytoplasmic isoform (ncOGT). However, this band was absent in Percoll-purified mitochondria, indicating that mOGT is the predominant, if not the sole, isoform present in mitochondria. For OGA, a single band, running at 75 kDa was observed in the mitochondrial fraction, which corresponds to the expected molecular weight of the short OGA isoform (sOGA), also expressed in the nucleus^18^. Conversely, the full length OGA (fOGA) running at 130 kDa was absent from the mitochondrial fraction. (Fig. 1a).

To assess whether protein O-GlcNAcylation was increased in our reconstitution assay, crude mitochondrial fractions were lysed after exposure to UDP-GlcNAc in absence or presence of the O-GlcNAc inducer NButGT. Following denaturation, O-GlcNAc-modified proteins were stabilized and labelled with the fluorescence probe TAMRA using Click-iT chemistry (Fig. 1c and S1). Following TAMRA-mediated O-GlcNAc specific immunoprecipitation, proteins were separated by gel electrophoresis and visualized by fluorescence. Multiple bands were observed in immunoprecipitates from control mitochondria indicating a baseline level of protein O-GlcNAcylation (Fig. 1c). Importantly, staining intensity was consistently increased in NButGT-treated mitochondria indicating a broad and rapid rise in protein O-GlcNAcylation levels.

### Acute stimulation of O-GlcNAcylation triggers rapid changes in the mitochondrial O-GlcNAcylome

**Fig. 2:**
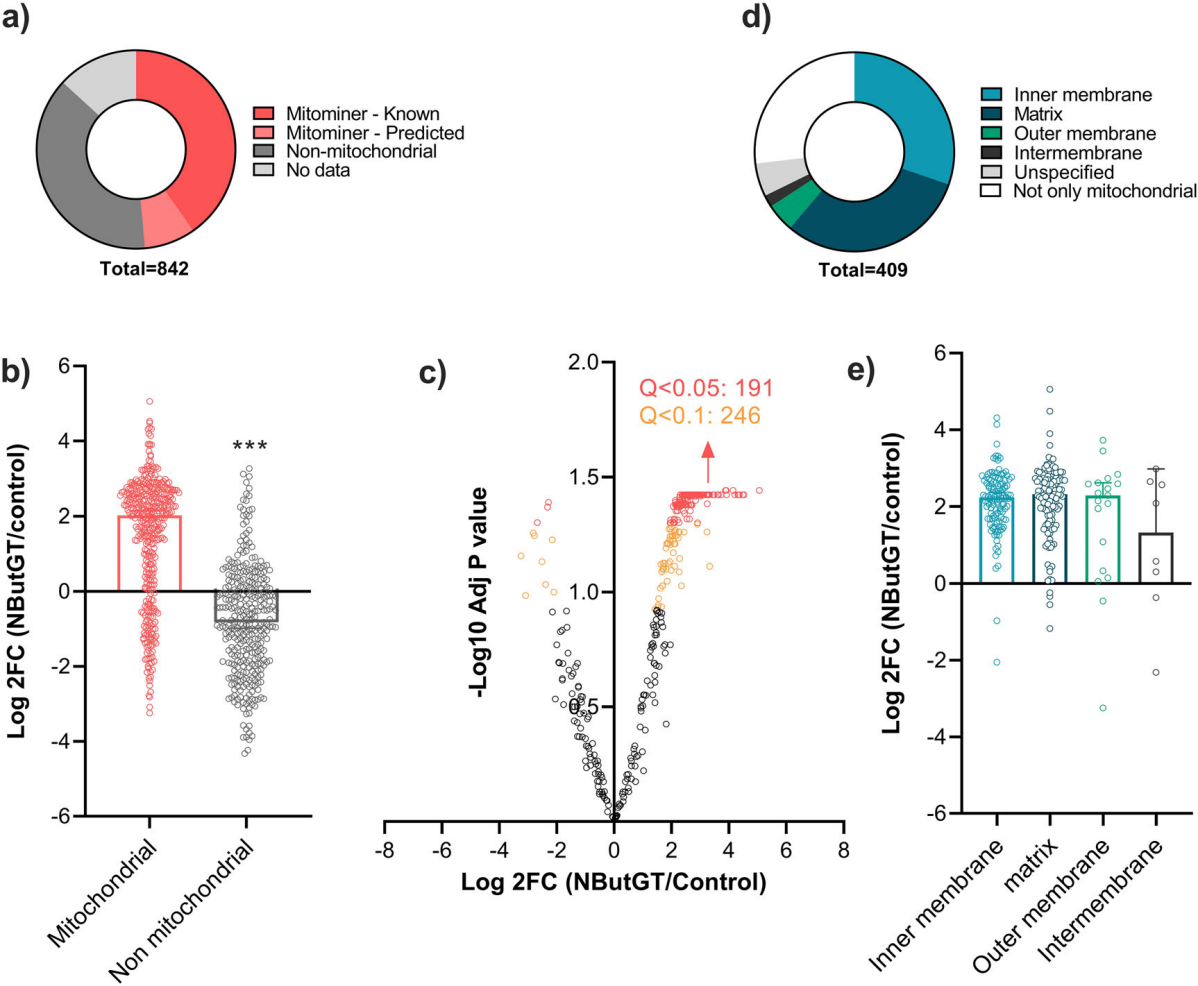
Impact of NButGT treatment on protein O-GlcNAcylation accordingly to their subcellular localization. **a** Relative distribution of proteins detected by MS/MS according to their status on the MitoMiner database. Only proteins reliably detected in all experimental replicates from control and NButGT-treated mitochondria were considered. **b** Fold change in the abundance of mitochondrial (*i.e*. known + predicted mitochondrial status) and non-mitochondrial O-GlcNAc-modified proteins between control and NButGT-treated mitochondria. **c** Volcano plot analysis showing the impact of NButGT O-GlcNAcylation for proteins with a mitochondrial status on the Mitominer database. Red (*q* < 0.05) and orange (*q* < 0.1) dots represent proteins that were significantly affected in response to NButGT. Number of significantly affected proteins are indicated along with the adjusted *p* value (q) threshold. Statistical analysis was assessed using a linear regression model (empirical Bayes methods) followed by the Benjamini-Hochberg FDR procedure. **d** Fold change in the abundance of O-GlcNAc-modified proteins between control and NButGT-treated mitochondria according to their sub-mitochondrial localization. **e** Relative distribution of mitochondrial proteins (known + predicted) according to their sub-mitochondrial localization. Proteins were ascribed to a particular sub-mitochondrial compartment based on annotations available in the Uniprot and GO databases. For panels **b**, **e**, individual values for each protein identified as well as mean ± sem (*n* = 3-4 biological replicates) are shown.

To gain knowledge on the repertoire of proteins modified by the mitochondrial O-GlcNAcylation system, gels were cut in seven bands of equal size and processed for tandem mass spectrometry (MS/MS) analysis. To maximize stringency, only proteins reliably detected in all experimental replicates from control and NButGT-treated mitochondria were considered. Using this selection criteria, a total of 842 proteins were identified (Fig. 2a). Of these, 50% (409) had a known (339) or predicted (70) mitochondrial status in the Mitominer database^19^, while the remaining were non-mitochondrial (322), or had an unspecified status (111), which can be expected given that crude mitochondrial fractions were used for this analysis. Since these likely contained residual amounts of ncOGT and fOGA outside mitochondria, we sought to determine whether the impact of NButGT on protein O-GlcNAcylation varied according to the localization of these proteins. As shown in Fig. 2b, treatment with NButGT predominantly increased O-GlcNAcylation of mitochondrial proteins (*i.e*. known + predicted mitochondrial status) compared to non-mitochondrial proteins, indicating that the reconstitution assay was effective at targeting the intra-mitochondrial O-GlcNAcylation system. Consistent with this notion, treatment with NButGT significantly increased O-GlcNAcylation of 191 (*q* < 0.05) to 246 (*q* < 0.1) mitochondrial proteins (Fig. 2c), while none of the non-mitochondrial proteins were significantly affected (Fig S2). Because mOGT was reported as preferentially associated with the mitochondrial inner membrane^13^, we looked at the sublocalization of mitochondrial proteins. This analysis indicated that a large proportion of the 409 O-GlcNAcylated mitochondrial proteins originated from the matrix (126) and inner-membrane (124), with only a minor proportion coming from the intermembrane space or outer membrane (Fig. 2d, e). However, the effect of NButGT on O-GlcNAcylation level did not differ significantly across submitochondrial compartments (Fig. 2e).

**Fig. 3:**
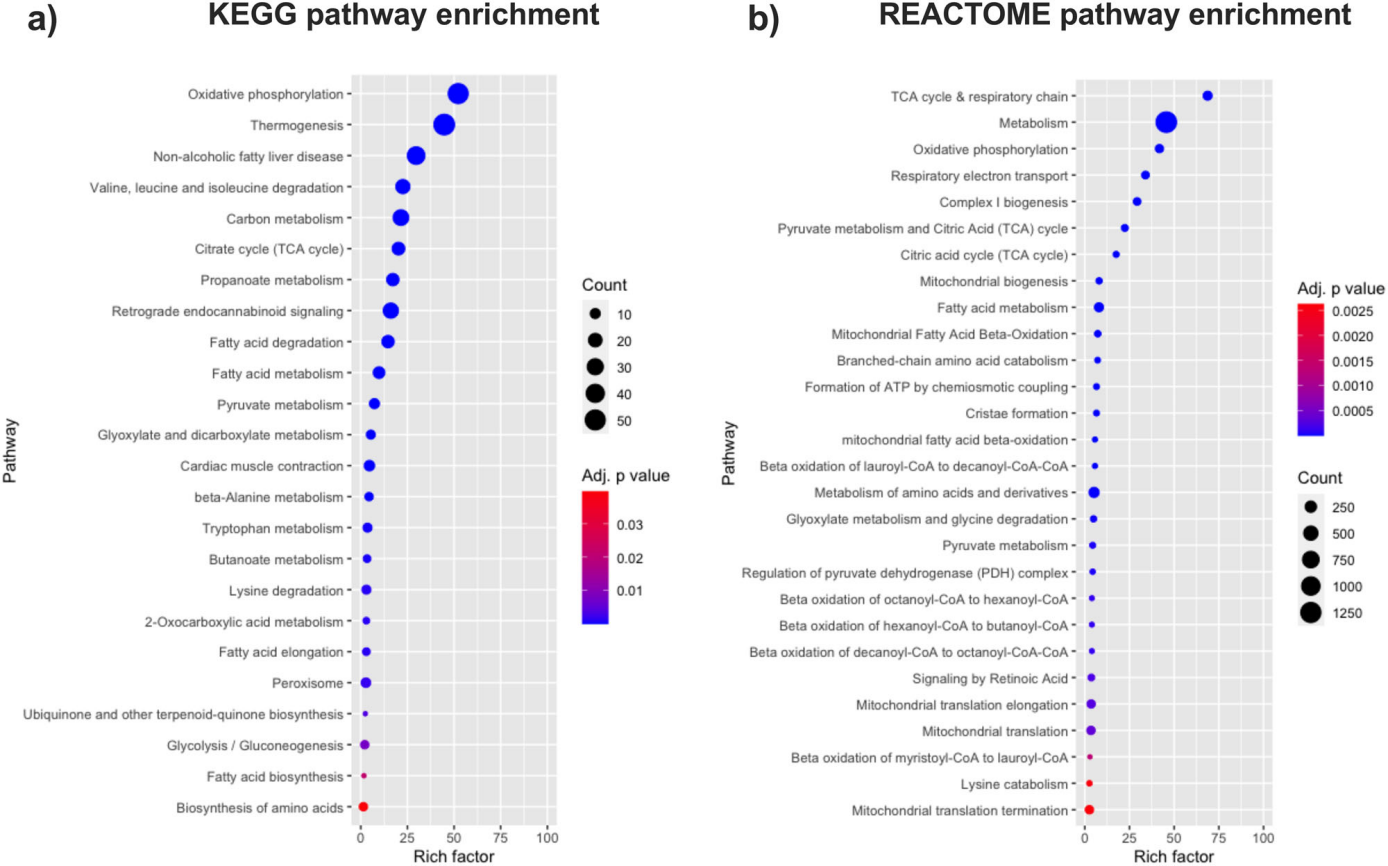
Characterization of the pathways over-enriched in response to NButGT in isolated mitochondria. Functional enrichment analysis of over-represented KEGG **a** and REACTOME **b** pathways in NButGT-treated vs control mitochondria performed using g:Profiler. Proteins were input in g:Profiler in order of decreasing *q* value with a threshold set at *q* < 0.1 (Ordered Query). Maximum size of functional categories was set at 250 to filter out large annotations that provide limited interpretative value. The g:SCS algorithm was used for multiple hypothesis testing corrections using a default alpha threshold of 0.05 for significance. Enrichment is expressed as a rich factor, which represents the ratio of the number of proteins observed for a given pathway term to the total number of proteins for this term. Circle size reflects the number of proteins per pathway, while colour indicates the level of significance.

**Fig. 4:**
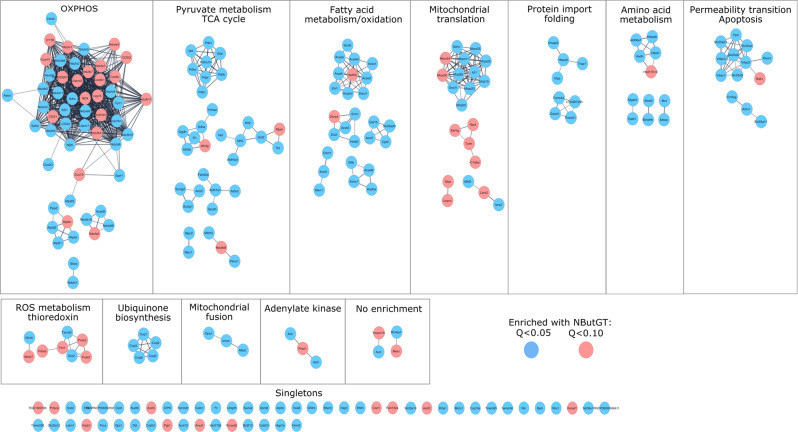
Characterization of the mitochondrial proteins displaying increased O-GlcNAcylation in response to NButGT in isolated mitochondria. High confidence (interaction score > 0.7 based on default active interaction sources) STRING network of mitochondrial proteins displaying increased O-GlcNAcylation in response to NButGT. Clustering was performed with the Markov Cluster (MCL) algorithm with a granular parameter set at 4. The Auto-annotate function of Cytoscape was used to identify pathways/processes corresponding to these clusters based on Stringdb descriptions and GO annotations. Proteins were color coded according to the q values smaller than 0.05 (blue) or 0.1 (red).

Based on these results, the mitochondrial processes targeted by acute mitochondrial O-GlcNAcylation were examined. Pathway enrichment analysis and protein network clustering revealed that proteins related to oxidative phosphorylation, tricarboxylic acid (TCA) cycle, and pyruvate and fatty acid metabolism were the top enriched pathways (Fig. 3a, b). Among the multi-proteins complexes of the oxidative phosphorylation machinery, complex I (23 subunits, 60%,), V (8 subunits, 50%), III (6 subunits, 75%), and II (3 subunits, 75%), were predominantly affected with 50–75% of their subunits being significantly more O-GlcNAcylated in response to NButGT compared to only 25% (4 subunits) for complex IV (Fig. 4). TCA cycle enzymes and pyruvate metabolism proteins displaying increased O-GlcNAcylation levels included isocitrate dehydrogenase (IDH2, IDH3A, IDH3B, IDH3G), aconitase (ACO2), succinyl-CoA ligase (SUCLG1, SUCLG2, SUCLA2), several subunits of the pyruvate dehydrogenase complex (PDHB, PDHX, PDHA1L1, DLAT, DLD) pyruvate carboxylase (PC), and both isoforms of the mitochondrial pyruvate carrier (MPC1-2). Besides, increased O-GlcNAcylated proteins of fatty acid metabolism included the carnitine-palmitoyl transferases and carnitine transporter (CPT1-2, SLC25A20), multiple β-oxidation enzymes (ACSL1, ACADS, ACADM, ACADL, ACADVL, DECR, ECHS1, ECI1, HADHA), and electron transferring flavoproteins (ETFA, ETFB).

Beyond energy metabolism, smaller clusters of proteins related to several other mitochondrial functions displayed increased O-GlcNAcylation levels in response to NButGT (Fig. 4). This included proteins related to: (i) mitochondrial protein translation, such as proteins associated with the mitochondrial ribosomes (MRPS11, MRPS22, MRPS23, MRPS25, MRPL22, MRPL38), (ii) protein processing such as proteases (CLPP, TRAP1), chaperones (HSPA9), and protein import channel subunits (TIMM21, TIMM44, SAMM50), (iii) proteins involved in the regulation of mitochondrial permeability transition such as cyclophilin-D (PPIF), voltage-gated anion channel, and ATP/ADP exchanger isoforms (DAC1-2, SLC25A5, SLC25A31) and (iv) ROS detoxifying systems including superoxide dismutase (SOD1-2), peroxiredoxins (PRX3, PRX5), thioredoxin (TXN1) and thioredoxin reductase (TXNRD2).

### The in vitro protein O-GlcNAcylation profile is reminiscent of protein O-GlcNAcylation observed following in vivo treatment with OGA inhibitors

**Fig. 5:**
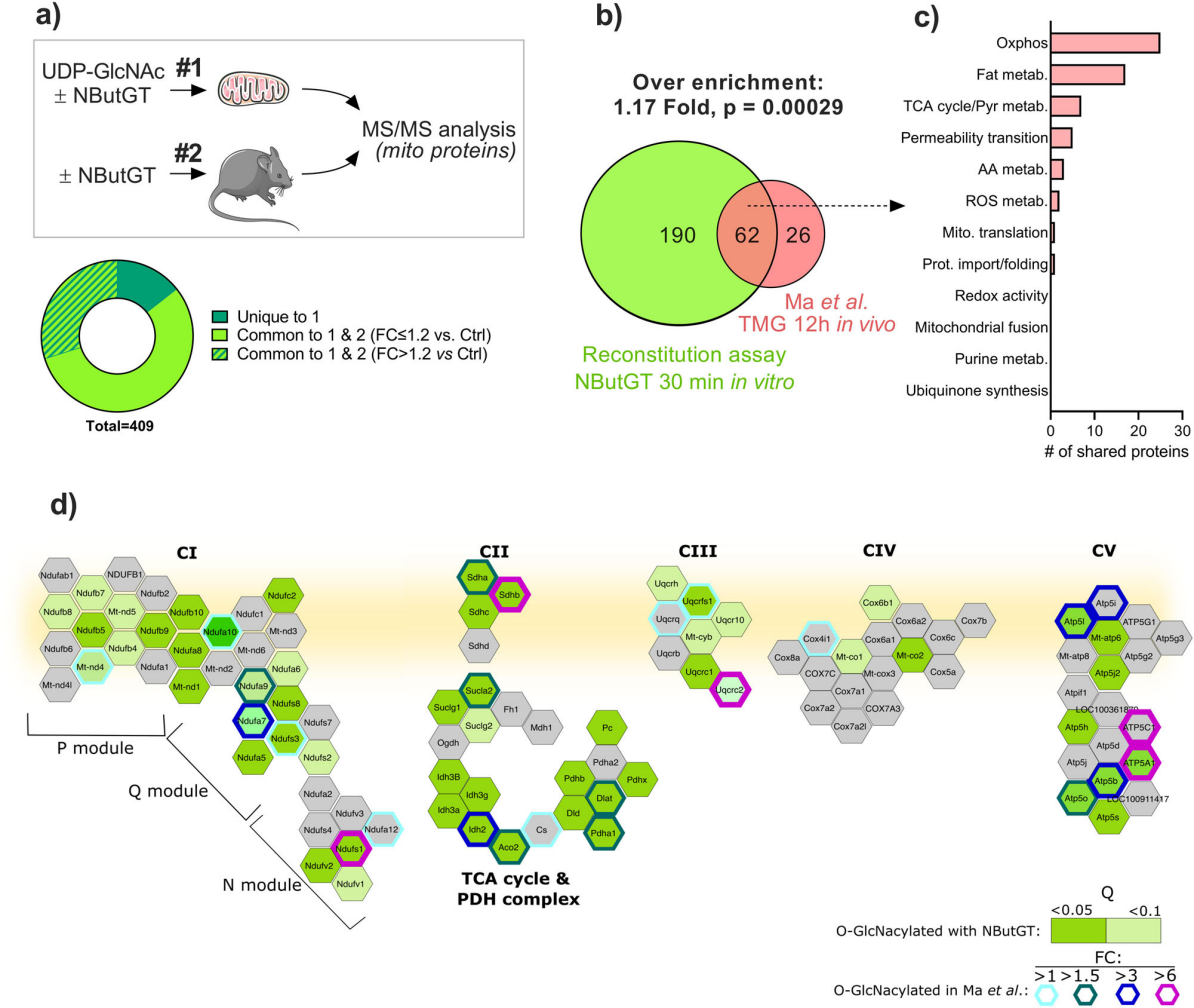
Comparison of mitochondrial protein O-GlcNAcylation profile following in vitro or in vivo OGA inhibition. **a** The dataset obtained following in vitro exposure of isolated mitochondria to NButGT (**#1**) was compared to a methodologically comparable (i.e. same Click-iT labelling, IP and MS/MS workflow) cardiac O-GlcNAcylomic dataset derived from mice that were treated with NButGT or vehicle 6 h prior to sacrifice (**#2**). The in vitro reconstitution dataset and the in vivo dataset contained 409 and 350 mitochondrial proteins, respectively. The circular chart indicates the proportion of proteins that were shared (light green) or unique (dark green) to the in vitro dataset. Hatched bar indicates shared proteins displaying sensitivity to NButGT with a FC cutoff > 1.2 vs. control. **b** Venn diagram showing the overlap between the in vitro reconstitution assay, and data from the Ma et al.^20^ study in which O-GlcNAc sites on isolated cardiac mitochondria were mapped 12 h after in vivo administration of the OGA inhibitor Thiamet G. Fold enrichment in the actual vs expected number of shared hyper-O-GlcNAcylated proteins between both datasets is shown along with the hypergeometric *p* value. For this test, the number of shared hyper-GlcNAcylated protein was compared to the total number of mitochondrial proteins identified in both datasets. **c** Number of shared proteins belonging to specific mitochondrial pathways/processes**. d** Overview of OXPHOS and TCA cycle proteins found to be hyper O-GlcNAcylated in the in vitro reconstitution assay and the Ma et al. dataset. Proteins labelled in grey were not detected.

To gain insights on the contribution of the intra-mitochondrial O-GlcNAcylation system to protein O-GlcNAcylation in the heart, we sought to compare these results with a methodologically comparable (*i.e*. identical Click-iT labelling, IP and MS/MS protocol) cardiac O-GlcNAcylomic dataset derived from mice that were subjected to NButGT or vehicle treatment 6 h prior to sacrifice (Fig. 5a). Of the 409 mitochondrial proteins detected in the in vitro reconstitution assay, 85% were also identified as being O-GlcNAcylated in vivo, and among them 122 displayed enhanced O-GlcNAcylation in the two data sets (FC > 1.2, Table S1).

Comparison was also made with data from a previous study performed by Ma et al. in which O-GlcNAc sites on isolated cardiac mitochondria were mapped 12 h after in vivo administration of the OGA inhibitor Thiamet G using a BEMAD labelling method^20^. Of the 88 O-GlcNAc-modified proteins identified by Ma et al., 62 were found to display increased O-GlcNAcylation in our in vitro reconstitution assay, representing a highly significant over-enrichment (Fig. 5b). The majority of shared proteins across the two datasets were components of the oxidative phosphorylation system, TCA cycle, and fatty acid oxidation pathway (Fig. 5c), with a few noticeable proteins related to ROS metabolism (SOD2, PRX3) and permeability transition pore (mPTP)/apoptosis (VDAC1, SLC25A4, ENDOG). Within the OXPHOS system, overlap between the two datasets was observed for subunits located in the NADH dehydrogenase (N) and ubiquinone reductase (Q) modules of complex I (NDUFS1, NDUFA7, NDUFA9), the F1 sector of complex V (ATP5O, ATP5B, ATP5A1), the hydrophilic head of complex II protruding in the matrix (SDHA, SDHB) and UQCRC2, a matrix facing subunit of complex III (Fig. 5d and S2).

**Fig. 6:**
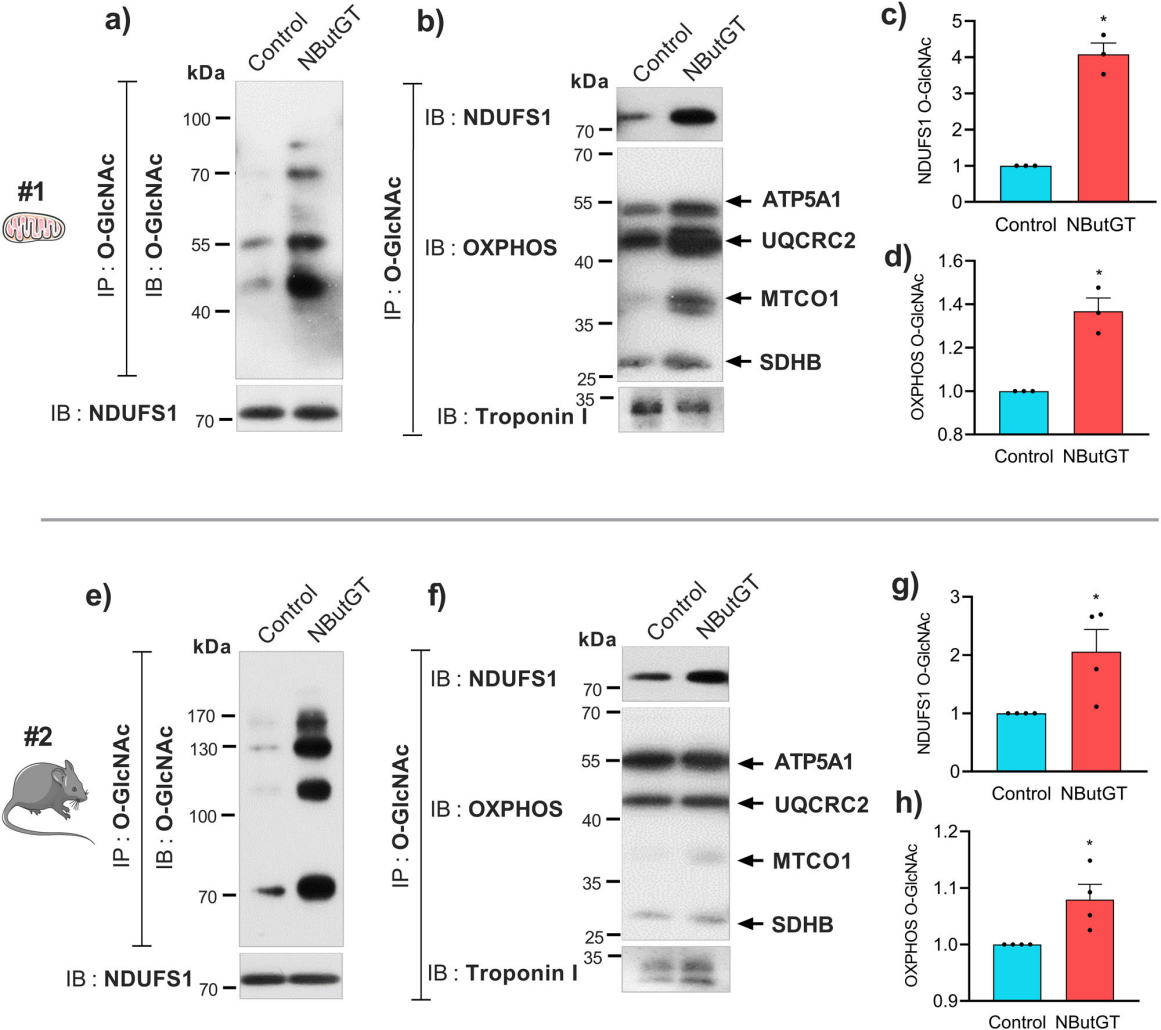
Validation of mitochondrial protein O-GlcNAcylation following in vitro or in vivo OGA inhibition. Mitochondrial and whole heart lysates used for experiment #1 (top panel) and #2 (bottom panel) were submitted to immunoprecipitation (IP) using an anti-O-GlcNAc antibody. Immunoprecipitations were confirmed using an anti-O-GlcNAc antibody in isolated mitochondria (**a**) and whole heart lysates (**e**). **b** Immunoprecipitates from isolated mitochondria were then immunoblotted (IB) with anti-NDUFS1, a cocktail of anti-OXPHOS antibodies, or an anti-Troponin I antibody. **c** Quantification of O-GlcNAc NDUFS1 following NButGT treatment in isolated mitochondria. **d** Quantification of O-GlcNAc OXPHOS following NButGT treatment in isolated mitochondria. **f** Immunoprecipitates from whole heart lysates were then immunoblotted (IB) with anti-NDUFS1, anti-OXPHOS set of antibodies or anti-Troponin I antibody. **g** Quantification of O-GlcNAc NDUFS1 following NButGT treatment in mice. **h** Quantification of O-GlcNAc OXPHOS following NButGT treatment in mice. Data are represented as means ± sem (3-4 biological replicates). Statistical comparisons were made using unpaired *t-tests*. *: *p* < 0.05. Uncropped gels with inputs and IP supernatants can be shown in Fig S6.

To validate our O-GlcNAcylomic data, immunoprecipitation of O-GlcNAcylated proteins was performed on lysates from control and NButGT-treated mitochondria using an anti-O-GlcNAc antibody, and the resulting immunoprecipitates were probed with antibodies directed against several identified proteins including NDUFS1, ATP5A1, UQCRC2, MTCO1, and SDHB. As shown in Fig. 6a, exposure to NButGT induced a strong increase in global mitochondrial O-GlcNAcylation, which was still conserved after immunoprecipitation. Furthermore, probing with the NDUFS1 antibody revealed a drastic increase in immunoreactivity following exposure to NButGT (Fig. 6b, c), without any changes in NDUFS1 protein abundance in the lysates (Fig. 6a). Similarly, O-GlcNAc staining of ATP5A1, UQCRC2, MTCO1, and SDHB (using the antibody mix OXPHOS) was increased following immunoprecipitation with the anti-O-GlcNAc antibody (Fig. 6b–d). To further validate the specificity of these results, the O-GlcNAc immunoprecipitates were also probed for a non-mitochondrial control protein (*e.g*. Troponin-I) present in the crude mitochondrial fraction, which was not affected by NButGT in the in vitro proteomics dataset (FC = 0.89, Adj P = 0.76). As shown in Fig. 6b, the O-GlcNAcylation level of Troponin I was not increased by NButGT in our in vitro assay.

These measurements were also performed in whole heart lysates from mice exposed to NButGT or vehicle in vivo. All OXPHOS subunits probed, including NDUFS1, displayed increased O-GlcNAcylation (Fig. 6f). In these experiments, the O-GlcNAcylation level of Troponin-I was also increased (FC 1.46 NButGT *vs*. Control), which was consistent with our proteomics results and previous results showing that in vivo, treatment with OGA inhibitors efficiently enhances O-GlcNAcylation of cardiac cytoplasmic proteins^8^.

### Acute stimulation of O-GlcNAcylation enhances maximal ETC flux and complex I activity

**Fig. 7:**
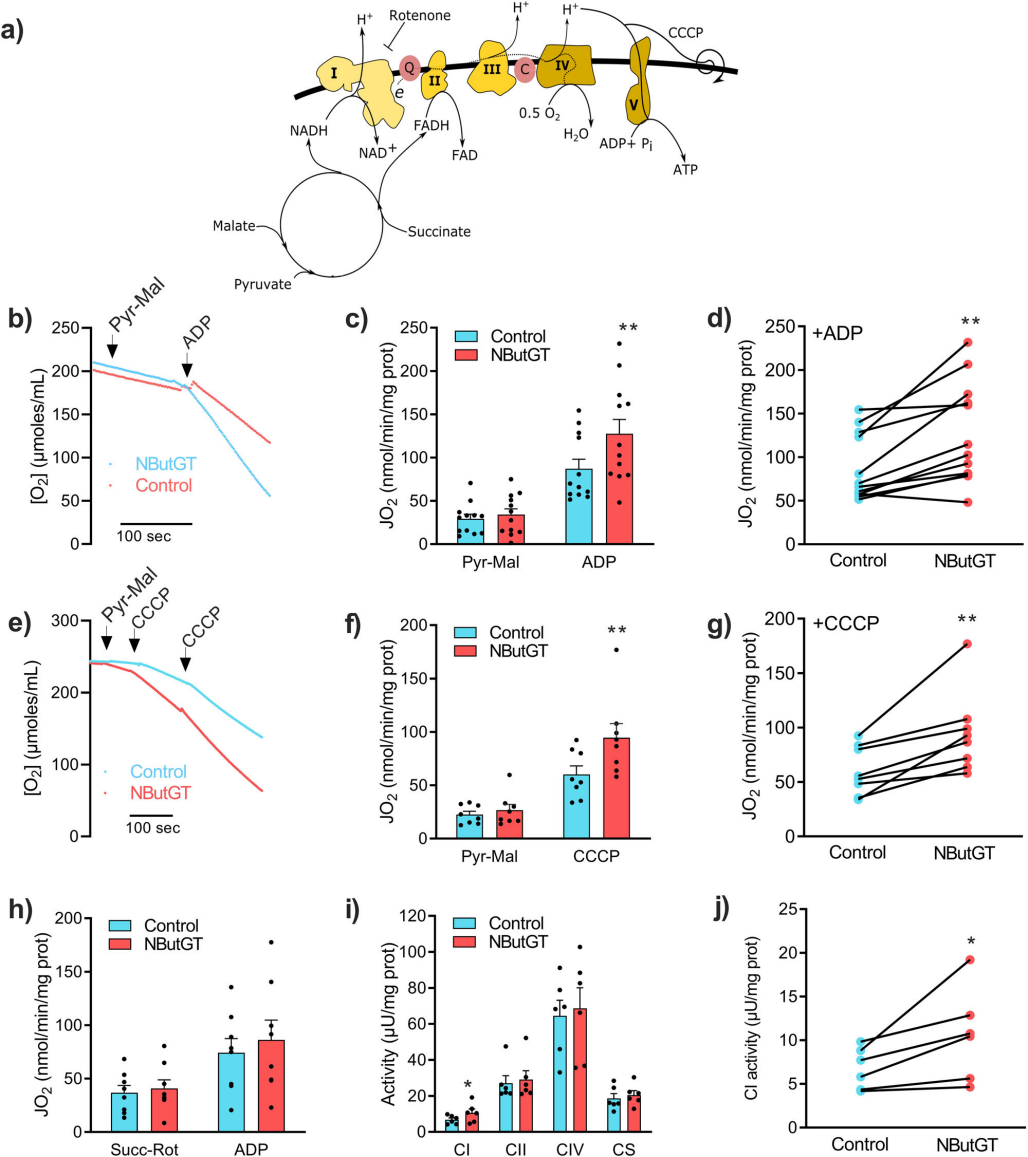
Impact of acute stimulation of mitochondrial O-GlcNAcylation on respiratory function. **a** Schematic illustration of the different substrates and inhibitors with their action sites. Following pre-incubation with UDP-GlcNAc in absence or presence of NButGT, mitochondria were transferred to respirometry chambers for the recording of baseline state 2 (St. 2), maximal ADP-stimulated (ADP), and CCCP uncoupled respiration in presence of complex I (Pyruvate-Malate [Pyr-Mal]) or complex II (Succinate in presence of the complex I inhibitor rotenone [Succ-Rot]) substrates. For all experiments, control and NButGT-treated mitochondria were tested in parallel, allowing pairwise comparisons. Panels **b**, **e** show representative respirometry tracings. Panels **c**, **f**, **h** show the calculated means ± sem for each respiratory state in the two experimental groups. Panels **d**, **g** illustrate the effect of NBuGT on ADP stimulated (**d**) or CCCP uncoupled respiration (**g**) for each of the paired incubations (*n* = 3 biological replicates with 2–3 technical replicates per group). **i** Enzyme activity of complex I, II, IV (CI, CII, and CIV) and citrate synthase (CS) measured spectrophotometrically in whole mitochondrial lysates. Data are represented as means ± sem (*n* = 3 biological replicates, 2 technical replicates). **j** Effect of NBuGT on complex I activity for each of the paired incubations performed. Statistical comparisons were made using paired two-sided t tests. *p < 0.05, **p < 0.01.

To determine whether such acute stimulation of O-GlcNAcylation had a functional impact, mitochondria were pre-incubated with NButGT or vehicle during 30 min before monitoring basal, ADP stimulated, and CCCP-uncoupled respiration (for the reader’s convenience, the different substrates and inhibitors with action sites is represented in Fig. 7a). As shown in Fig. 7b–d, exposure to NButGT increased maximal ADP-stimulated respiration in presence of TCA cycle substrate feeding predominantly complex I (pyruvate – malate). A similar effect was observed when phosphorylation was uncoupled from respiration using CCCP (Fig. 7e–g), which suggests that activation of the electron transport chain (ETC), plays an important role in stimulating OXPHOS capacity. Interestingly, NButGT had no significant effect on ADP-stimulated respiration when mitochondria were energized with complex II substrate (succinate in presence of rotenone), suggesting that the stimulatory effect of NButGT was linked to complex I, although stimulation of pyruvate transport in mitochondria or TCA cycle dehydrogenases could also contribute (Fig. 7h). This was directly confirmed by measuring the activity of respiratory chain complexes in mitochondrial lysates. Following exposure to NButGT, the activity of complex I was increased by ~ 50%, while those of complex II, complex IV, and the TCA cycle enzyme citrate synthase were unchanged (Fig. 7i, j). Of note, respirometry experiments were also performed in presence of the other O-GlcNAc inducer Thiamet G (also an OGA inhibitor), and yielded comparable results (Fig S3). Altogether, these data provided evidence that O-GlcNAcylation increases maximal electron flux through complex I. Furthermore, since the complex I assay measures electron transport from NADH to ubiquinone via the FMN and Fe-S redox centres^21^, these results provided evidence that at least some of the putative O-GlcNAcylation sites underlying this stimulatory effect were located in the N and Q modules of complex I.

### Acute stimulation of O-GlcNAcylation attenuates mitochondrial ROS release

To determine whether this had an impact on ROS release, H_2_O_2_ production was determined in mitochondria that were pre-incubated with NButGT, Thiamet G, or vehicle. H_2_O_2_ release was first measured at baseline in presence of pyruvate and malate.

**Fig. 8:**
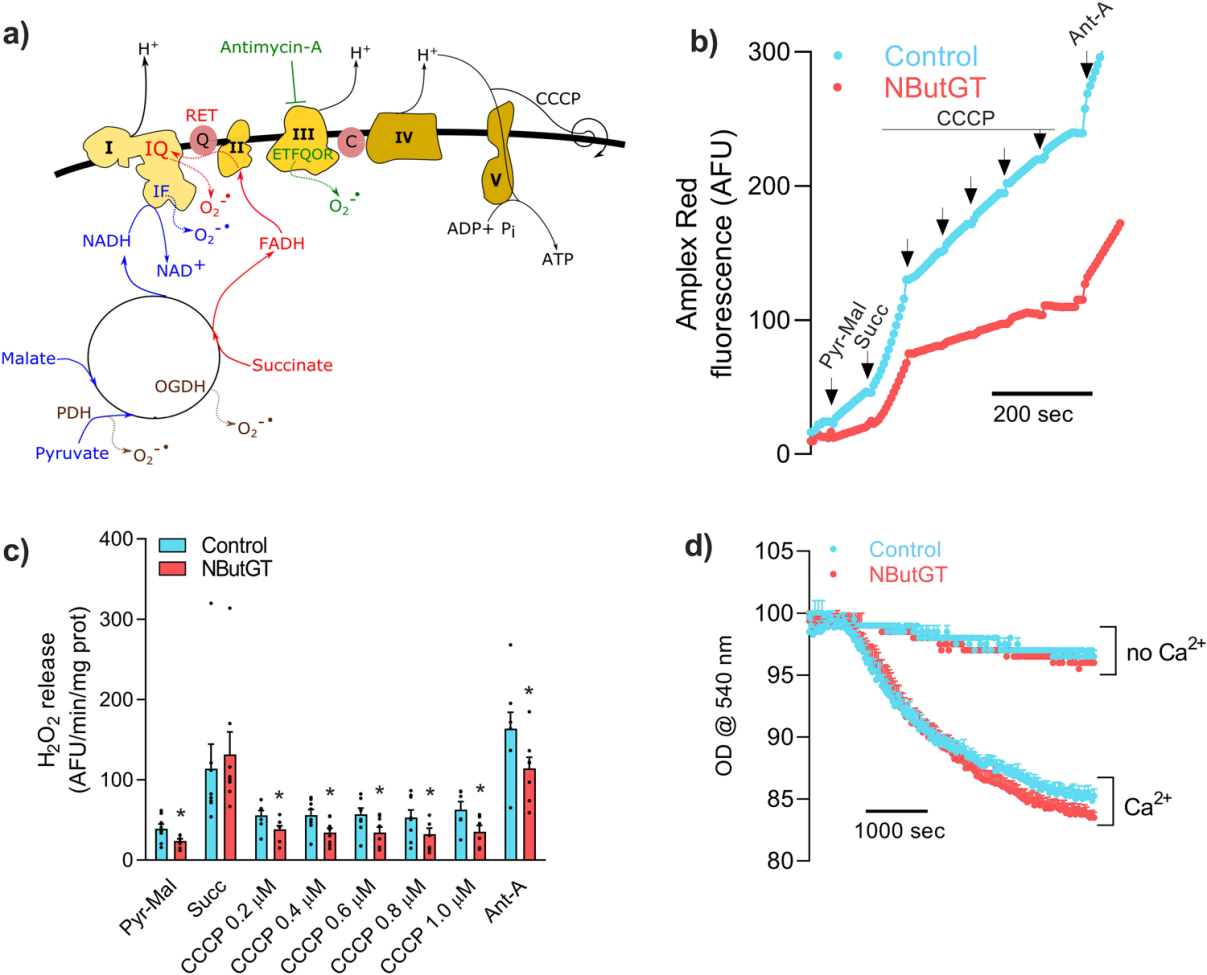
Impact of acute stimulation of mitochondrial O-GlcNAcylation on ROS release and sensitivity to Ca^2+^-induced permeability transition. **a** Schematic illustration of the different substrates and inhibitors with the electron flux. Following pre-incubation with UDP-GlcNAc in absence or presence of NButGT, mitochondrial H_2_O_2_ release was measured following sequential addition of the complex I substrates pyruvate-malate (Pyr-Mal), the complex II substrate succinate (Succ), the uncoupler CCCP, and the complex III inhibitor Antimycin-A (Ant-A). For all experiments, control and NButGT-treated mitochondria were tested in parallel, allowing pairwise comparisons. **b** Representative Amplex Red fluorescence tracing of control and NButGT treated mitochondria. **c** Rate of H_2_O_2_ emission calculated under the various respiratory states in two experimental groups. Data are represented as means ± sem (*n* = 4 biological replicates with 2 technical replicates per group). **d** Sensitivity to Ca^2+^-induced permeability transition pore opening was assessed by monitoring swelling at 540 nm. A *t* = 0 s CaCl_2_ (150 µM) was added to mitochondria energized with pyruvate and malate and absorbance changes were monitored during 60 min. Data are represented as means ± sem (*n* = 3 biological replicate with 2-3 experimental replicates per experimental group). Multiple t-tests were used to establish statistical significance. *p < 0.05, corresponding to *q* value of <0.1.

As represented in Fig. 8a, under this condition (eg pyruvate-malate), electron flux through the ETC is in the forward direction, and ROS originate from the N module of complex I *i.e*. the FMN and Fe-S clusters of complex I (IF site; ~30% contribution), the electron-transferring flavoprotein ubiquinone reductase site within complex III (ETFQOR; ~30 % contribution), and TCA cycle enzymes (*e.g*. OGDH and PDH, ~40% contribution)^22^. H_2_O_2_ release under this condition was decreased by more than 40–50% in NButGT (Fig. 8b, c) or Thiamet G-treated (Fig S4) mitochondria compared to controls, suggesting that one or more of these ROS release sites were affected by O-GlcNAcylation.

The complex II substrate succinate was next added to elicit a dual influx of electrons in the ETC, and to stimulate reverse electron transport (RET) from complex II to complex I. Under this condition, a large proportion (*i.e*. ~80%) of ROS originates from the Q module of complex I (IQ) as a result of RET (Fig. 8a)^22,23^. To a lesser extent, ROS release through the IF site of CI and ETFQOR of CIII can also contribute (~10% each). Interestingly, no difference in H_2_O_2_ release was observed between control and NButGT (Fig. 8b, c) or Thiamet G-treated (Fig S4) mitochondria under this condition, suggesting that ROS release through RET at the IQ site of complex I was not reduced by O-GlcNAcylation.

To test this further, mitochondria were progressively depolarized with the uncoupler CCCP since RET, and therefore ROS release by the IQ site, is exquisitely sensitive to the electrochemical gradient^24^. As expected, abolishing RET through uncoupling caused a drastic reduction of H_2_O_2_ release from the IQ site, and restored the 40-50% difference in H_2_O_2_ release observed between control and NButGT- or Thiamet G-treated mitochondria in absence of RET (Fig. 8b, c and S3).

The ETFQOR site within complex III represents a major site of ROS production in the ETC. In isolated mitochondria, this site dominates superoxide release from the ETC particularly when electron transfer is blocked with complex III inhibitors (Fig. 8a)^24^. For this reason, antimycin-A was next added to fully uncoupled mitochondria in order to further assess the impact of NButGT on ROS release from complex III. As expected, addition of antimycin-A caused a drastic rise in H_2_O_2_ release reflecting superoxide release from complex III in all groups (Fig. 8b, c). In NButGT-treated mitochondria, H_2_O_2_ release was reduced by 30% compared to controls suggesting reduced release of ROS from the ETFQOR site (Fig. 8b, c). However, this effect was not observed in presence of Thiamet G (Fig S4), suggesting that the two compounds exert slightly different effects on complex III subunits. Overall, these results suggest that hyper O-GlcNAcylation causes a reduction of mitochondrial ROS release likely through mechanisms implicating ETFQOR of CIII, the IF site of CI, and possibly subunits of OGDH and PDH, which were significantly hyper-O-GlcNAcylated following exposure to NButGT (Fig. 5d).

Of note, previous studies reported that increased cellular O-GlcNAcylation protects from mitochondrial permeability transition pore (mPTP) opening^14,20^. Mitochondrial swelling assays were therefore performed to determine whether sensitivity to Ca^2+^-induced mPTP opening was altered following acute exposure to NButGT. As shown in Fig. 8d, although proteins associated with the regulation of the mPTP were found to be O-GlcNAcylated (see Fig. 8d), acute inhibition of mitochondrial OGA had not effect on the sensitivity to permeability transition.

## Discussion

O-GlcNAcylation of mitochondrial proteins was previously shown to be responsive to inhibition of O-GlcNAc cycling enzymes (OGT/OGA)^13,25^ as well as to altered intracellular UDP-GlcNAc levels^26^. However, controversy existed regarding the precise mechanism leading to mitochondrial protein O-GlcNAcylation. Moreover, the repertoire of proteins affected as well as the functional impact remained ill-defined. Taking advantage of our in vitro reconstitution assay, which allows to isolate mitochondria from non-mitochondrial O-GlcNAc cycling systems, our study shows that O-GlcNAc cycling enzymes are present in functionally relevant amounts in the mitochondrial compartment and can trigger broad and rapid changes in protein O-GlcNAcylation which are highly reminiscent of the mitochondrial O-GlcNAcylation profile observed in vivo. Our proteomic workflow confirms previously reported O-GlcNAc-modified proteins, and identifies several novel targets related to energy metabolism, and multiple other facets of mitochondrial biology. Importantly, we show that acute hyper-O-GlcNAcylation increases maximal respiratory capacity, and drastically reduces ROS release.

The OGT gene encodes three splice variants whose products vary only in the number of N-terminal tetratricopeptide repeat (TPR) domains known to be involved in protein-protein interaction^1^. The longest splice variant encodes the 116 kDa nucleocytoplasmic ncOGT isoform which is the most abundantly expressed, while the shortest 78 kDa isoform is curiously derived from a longer transcript. In addition, Hanover’s group identified a unique start site in the fourth intron of the OGT gene that generates a 103 kDa isoform which was found to be enriched in the mitochondrial fraction of Hela cells^15,16^ and rat heart^13^. However, the existence of this mitochondrial isoform in mammalian tissues has been recently questioned^17^. In this study, endogenous mOGT was reported to be undetectable in several human cell lines and mouse tissues, including heart. Genomic sequence alignments also suggested that the predicted start site for mOGT was likely lacking in most species analyzed except some primates. Based on these data, the authors concluded that the small amounts of ncOGT detected in the crude mitochondrial fraction was likely sufficient for O-GlcNAcylation of mitochondrial proteins. These results are however in contrast with our data and previous studies from Hanover’s group. Our data clearly indicate that the 103 kDa mOGT is the main isoform found in Percoll-purified cardiac mitochondria. Comparison between crude and Percoll-purified mitochondrial preparations in fact suggests that the small amount of ncOGT found in the crude fraction is an extra-mitochondrial contaminant. Beside mOGT, our results also establish that the nuclear-predicted sOGA isoform^18^ is the sole OGA isoform present in mitochondria. The fact that inhibition of OGA with NButGT specifically increased O-GlcNAcylation of mitochondrial proteins, without affecting non-mitochondrial proteins remaining in the reconstitution assay, provides further support for the mitochondrial localization of sOGA.

Isolated mitochondrial preparations have been used previously to study UDP-GlcNAc uptake kinetics, which has led to the identification of the pyrimidine nucleotide carrier (SLC25A33) as the main transporter for UDP-GlcNAc^13^. However, the functional coupling between the uptake of UDP-GlcNAc and the intramitochondrial O-GlcNAc cycling enzymes has never been examined. Our data suggest that UDP-GlcNAc is transported in mitochondria in sufficient amounts to allow changes in protein O-GlcNAcylation levels by NButGT treatment. Using our reconstitution assay, we were able to map a large proportion of previously identified O-GlcNAcylated proteins^20^ involved in energy metabolism. Collectively these results confirm that proteins of the OXPHOS machinery, TCA cycle, and fatty acid metabolism pathways are prominent targets for O-GlcNAcylation. In addition, we identified several other proteins, including proteins involved in mitochondrial protein translation, protein import/chaperoning, and ROS/Redox homoeostasis. Future studies may reveal a role for these proteins in the cardioprotective effect of hyper-O-GlcNAcylation in the context of ischaemic injury^6^, and sepsis^9,10^.

Although O-GlcNAcylation of mitochondrial proteins has been associated with changes in function such as respiration, ROS release, and Ca^2+^-induced permeability transition^11^, the mechanisms remain largely elusive. The present study provides insights on potential links between functional changes and O-GlcNAcylation of specific proteins of the ETC and TCA cycle.

Enzyme activity measurements combined to functional assays revealed that complex I is likely one of the functionally relevant sites of regulation OXPHOS and H_2_O_2_ release by O-GlcNAcylation. The N module of complex I consists of several core catalytic subunits containing the FMN prosthetic groups, iron–sulfur clusters, as well as several accessory subunits which altogether oxidize NADH and transfer electrons to the Q and P modules to support proton pumping. Our O-GlcNAcylomic data revealed that subunits within this module displayed greater increases in O-GlcNAcylation compared to other complex I subunits. Similarly, in the Ma et al.^20^ dataset, one of the subunits displaying the highest fold change following in vivo treatment with Thiamet G was NDUFS1, located in the N module. This subunits, which was also hyper-O-GlcNAcylated in our dataset, harbours an NADH-ubiquinone oxidoreductase domain and Fe-S clusters, in addition to containing a number of phosphorylation^27^ and acetylation sites, which makes it a potentially important candidate for post-translational regulation of complex I activity. Along the same line, phosphorylation of four other subunits within the N module (NDUFS4, NDUFV1, NFUFV3, and NDUFA12), including one (NDUFSV1) that displayed sensitivity to NButGT, were previously shown to regulate complex I activity and ROS release^28–30^. As for the Q module, it encompasses several subunits collectively involved in the reduction of ubiquinone, including NDUFA7 and NDUFA9 which were identified as being O-GlcNAcylated in our dataset, and in the study by Ma et al.^20^. Interestingly, recent data indicate that inactivation of NDUFA7 in the heart increases mitochondrial ROS release, and triggers pathological cardiac hypertrophy^31^, although it is still unclear whether this is linked to failed assembly of complex I or to altered activity. Further studies will clearly be required to fully elucidate the mechanism by which acute O-GlcNAcylation stimulates complex I activity and contributes to modulate respiratory capacity and ROS release.

In addition to complex I, our data suggest that complex III may constitute another regulatory relevant site affecting ROS release. Our O-GlcNAcylomics data revealed that several subunits of complex III were significantly hyper O-GlcNAcylated following treatment with NButGT (UQCRH, UQCRFS1, UQCR10, MT-CYB, UQCRC1 and UQCRC2). Among those, three were also hyper O-GlcNAcylated in the Ma et al. dataset (UQCRFS, UQCRQ, and UQCRC2). One possibility is thus that O-GlcNAcylation of one or more of these subunits lowers electron leaks at the ETFQOR site.

In addition to these ETC complexes, we cannot exclude the possibility that enhanced respiration and reduced ROS release in presence of pyruvate-malate are also at least partly related to O-GlcNAcylation of other targets such as proteins involved in the TCA cycle. For instance, our O-GlcNAcylomics data revealed that the dihydrolipoamide acyltransferase (DLAT or E2) and dihydrolipoamide dehydrogenase (DLD or E3) subunits of OGDH and PDH were among the proteins found to be hyper-O-GlcNAcylated following exposure to NButGT. Superoxide/H_2_O_2_ production by OGDH and PDH was shown to be substantial in presence of their respective substrates (e.g. 2-oxoglutarate and pyruvate), accounting for up to 40 % of mitochondrial ROS release under state 2 conditions^22^. In the present study, the impact of O-GlcNAcylation on the activity of these enzymes was not examined. Nevertheless, our data suggests that O-GlcNAcylation of E2/E3 of PDH involved in pyruvate catabolism, could contribute to lower mitochondrial ROS release.

Although in vitro reconstitution assays are well suited for mechanistic studies, they pose obvious limitations. Firstly, a 30-min pre-incubation with UDP-GlcNAC and OGA inhibitors is required to observe functional effects, which is in line with previous transport experiments using ^3^H-UDP-GlcNAC in isolated cardiac mitochondria showing that 30 min are required to reach peak UDP-GlcNAc concentrations in the matrix^13^. As this is associated with a certain degree of functional decline due to “in vitro aging”, an alternative interpretation of the present data is that acute O-GlcNAcylation could protect mitochondria from functional inactivation during incubation at room temperature. However, irrespective of whether NButGT increases ETC flux or prevents functional inactivation, the mechanisms put forth in this study remain valid: acute O-GlcNAcylation enhances Complex I (CI) activity, increases maximal respiration mainly in presence of pyruvate and malate, and lowers mitochondrial ROS release.

Another limitation of in vitro reconstitution assays is that they do not fully mimic the complex conditions encountered in vivo. To tackle this issue, we compared our in vitro data with a methodologically comparable cardiac O-GlcNAcylomic dataset derived from mice injected with NButGT for 6 h before cardiac isolation. Our results reveal a strikingly high degree of overlap (85%) in the O-GlcNAc modified mitochondrial protein identified in the two datasets. Importantly, 122 of these proteins also displayed sensitivity to NButGT. Besides, highlighting the relevance of our reconstitution assay for mechanistic studies, these results highly suggest that the intra-mitochondria O-GlcNAc cycling system is the main mechanism through which mitochondrial proteins become O-GlcNAcylated in vivo.

## Methods

### Animal care

For in vitro studies, all experiments on animals were approved by the University of Ottawa Institutional Animal Care Committee and conducted according to the directives of the Canadian Council on Animal Care. Eight week-old rats (Wistar, male) were euthanized by thoracotomy following ketamine‐xylazine anaesthesia. For in vivo dataset, experiments were approved by the Animal Research Committee of the Université catholique de Louvain and conformed to the American Heart Association Guidelines for Use of Animal in Research. Twelve week-old mice (C57BL/6 N, male) from Janvier Labs, housed with a 12 h/12 h light/dark cycle, had free access to water and standard chow.

### Preparation of isolated cardiac mitochondria

Heart mitochondria were prepared as described previously^32^. Hearts were rapidly excised and immersed into ice-cold isolation medium (buffer A, in mM: 300 sucrose, 10 Tris–HCl, 1 EGTA, pH 7.3) and weighed. Ventricular tissue was minced with scissors in 5 ml of buffer A supplemented with 0.2% fatty acid free bovine serum albumin (BSA) and homogenized using a Polytron tissue tearer (~3 s at a setting of 3). The homogenate was then incubated with the protease Nagarse (1.5 mg/g) for 5 min and further homogenized at the same settings. The homogenate volume was completed to 30 ml with Buffer A + 0.2% BSA and centrifuged at 800×g for 10 min. The pellet was discarded and the supernatant was decanted and centrifuged at 10 000×g for 10 min. The pellet obtained was re-suspended in buffer B (in mM: 300 sucrose, 0.05 EGTA, 10 Tris–HCl, pH 7.3) and centrifuged at 10,000×*g* for 10 min. After repeating this washing step twice, the final mitochondrial pellet was re-suspended in 0.3 ml of buffer B to a protein concentration of ~ 20 mg/ml. All procedures were carried out at 4 °C. Protein determinations were performed using the bicinchonic acid method (Pierce, Rockford, IL, USA), with bovine serum albumin as a standard.

### In vitro reconstitution assay

Mitochondria (2 mg/mL) were incubated at room temperature during 30 min in buffer C (in mM: 250 sucrose, 10 MOPS, 0.005 EGTA, 2 KH_2_PO_4_, 0.2 MgCl2, pH 7.2) containing UDP-GlcNAC in presence of the OGA inhibitor NButGT or its vehicle (H_2_O). In some experiments NButGT was replaced by Thiamet G or its vehicle (H_2_O). After 30 min, mitochondrial suspensions were used for functional analyses as described below. Alternately, samples were centrifuged at 10,000×*g* for 10 min. 20 µL of supernatant was left and concentrated mitochondrial pellets were immediately frozen in liquid nitrogen for MS/MS analysis. The duration of the pre-incubation period, and the concentration of substrates and inhibitors used were selected based on previously published evidence and preliminary testing. More specifically, previous transport experiments using ^3^H-UDP-GlcNAC in isolated cardiac mitochondria have shown that 30 min are required to reach peak UDP-GlcNAc concentrations in the matrix^13^. Preliminary experiments also confirmed that UDP-GlcNAc and NButGT had no immediate effects on respiration when added alone or in combination, which further re-enforced the need for a pre-incubation period. As for the concentration used for UDP-GlcNAc, it was set at 2 mM, to insure an adequate supply for mOGT, which has a lower affinity for UDP-GlcNAc than ncOGT^33^. This concentration is ~4 fold higher than the reported Km of the Pnc1 transporter (400–500 µM)^34^ involved in the uptake of UDP-GlcNAC in mitochondria^13^. As for the two OGA inhibitors, concentrations shown to be effective in cell culture studies were used, namely 10 µM for NButGT and 5 µM for ThiametG^8,9,35–39^.

### Respirometry

Mitochondria (0.5 mg of protein) were incubated at room temperature in 1 ml of buffer C in Hansatech respirometry chambers. After recording baseline oxygen consumption, ADP-restricted state 2 respiration was measured in presence of TCA cycle substrates feeding predominantly CI (Pyruvate-Malate 5: 2.5 mM) or complex II (Succinate + Rotenone 5 mM: 1 µM). ADP (1 mM) or CCCP (0.4 µM) was then added to elicit maximal ADP-stimulated or uncoupled respiration respectively^32^. Prior to these experiments, titrations with CCCP were performed in freshly isolated mitochondria to select the concentration eliciting full uncoupling.

### Mitochondrial H_2_O_2_ release

Net H_2_O_2_ release by respiring mitochondria was measured fluorimetrically using the H_2_O_2_ sensitive probe Amplex red (excitation-emmission: 563–887 nm) as previously described^40^. Mitochondria (0.5 mg/mL) were incubated in 600 µL of buffer Z at 37 °C (in mM: 110 K-Mes, 35 KCl, 1 EGTA, 5 K2HPO4, 3 MgCl26H2O and 0.5 mg mL^−1^ BSA, pH 7.3 at 4 °C) containing HRP (1.2 U/mL), and Amplex Red (5 µM). Baseline fluorescence readings were taken in the absence of any exogenous respiratory substrates. The following additions were then made sequentially: Pyruvate-malate (5:2.5 mM), succinate (5 mM), CCCP (0.2, 0.4, 0.6, 0.8, 1.0 µM) and Antimycin-A (8 µM). Rates of H_2_O_2_ release were calculated by measured the slopes of change in Amplex red fluorescence and reported in arbitrary fluorescence units.

### Mitochondrial swelling assay

Sensitivity to opening of the permeability transition pore (PTP) was determined spectrophotometrically on a plate reader by monitoring osmotic swelling in response to calcium. Mitochondria (0.5 mg/mL) were incubated in buffer D (in mM: 250 sucrose, 10 MOPS, 0.005 EGTA, 10 KH_2_PO_4_, pH 7.2, 37 °C) supplemented with 5 mM Pyruvate, and 2.5 mM Malate. Following recording of baseline absorbance at 540 nm, a single pulse of 150 µM CaCl_2_ was added in each well and changes in absorbance were monitored during 45 min. Changes in absorbance were expressed relative to baseline values for each well, and onset of swelling was taken as indicator of PTP sensitivity.

### Enzyme activities

Activities of complex I (NADH-CoQ reductase), complex II (succinate dehydrogenase), complex IV (cytochrome oxidase), and citrate synthase were measured spectrophotometrically in a plate reader using standard coupled enzyme assays adapted from^32^. Mitochondria were disrupted in a lysis buffer (in mM: KCl 120, HEPES 20, MgCl2 2, EGTA 1, with 5 mg/ml BSA, Ph 7.4) using 3 freeze-thaw cycles and 10 sec sonication of 40% maximal intensity. Protein concentration was determined using the BCA assay and were kept on ice until use. Determinations were made in triplicate for each sample using 0.25 (CS and CIV) or 2 µg (CI, CII) of proteins per well. For complex I, the assay was performed at 340 nm using the acceptor 2,3-dimethoxy-5-methyl-6-*n*-decyl-1,4-benzoquinone (80 μM) and 200 μM NADH as donor, in 10 mM Tris (pH 8.0) buffer containing 1 mg/ml BSA, 0.24 mM KCN and 0.4 μM antimycin-A for 5 min. The addition of 4 μM rotenone to each well allowed us to quantify the rotenone-sensitive activity. For complex II, the assay was performed at 600 nm using the acceptor DCPIP (80 μM) and 10 mM succinate as the donor in a medium containing 10 mM KH_2_PO_4_ (pH 7.8), 2 mM EDTA, 1 mg/ml BSA in the presence of 80 μM 2,3-dimethoxy-5-methyl-6-*n*-decyl-1,4-benzoquinone, 0.24 mM KCN, 4 μM rotenone, 0.2 mM ATP and 0.4 μM antimycin-A for 2 min. The addition of 10 mM malonate to each well inhibited the oxidation of succinate. For complex IV, the assay was performed at 550 nm using 10 μM reduced cytochrome *c* as donor in a isoosmotic medium (10 mM KH_2_PO_4_ (pH 6.5), 1 mg/ml BSA, 0.3 M sucrose) for 2 min, after permeabilizing both mitochondrial membranes with 2.5 mM *n*-duodecyl-β-D-maltoside. For citrate synthase, the assay was performed at 412 nm following the reduction of 2 mM 5,5′-dithio-bis(2-nitrobenzoic acid) in the presence of 0.1 mM acetyl-CoA and 12 mM oxalacetic acid in a medium with 10 mM KH_2_PO_4_ (pH 7.8) containing 2 mM EDTA, 1 mg/ml BSA and 0.1% Triton X-100. All activities were expressed in mU min^−1^ mg mitochondrial prot^−1^.

### In vivo NButGT treatment

Twelve week-old mice (C57BL/6 N, male) from Janvier Labs were treated with NButGT (50 mg/kg) by intraperitoneal injection 6 h before sacrifice. This concentration was previously shown by our group and others to be effective in inducing a global increase in cardiac protein O-GlcNAcylation^8,41^. NBuGT was dissolved in saline buffer (NaCl 0.9%) at a concentration of 10 µg/µL and ~150 µL was injected in mice. Mice were anesthetized with an intraperitoneal injection of a mixture of ketamine (150 mg/kg) and xylazine (10 mg/kg) and hearts were washed in PBS before being freeze-clamped in liquid nitrogen and stored at −80 °C.

### O-GlcNAc immunoblotting and immunoprecipitation

Lysate supernatants (20 µg of heart homogenate, nuclear fraction, cytosolic fraction and crude mitochondria; 40 µg of purified mitochondria) were loaded on SDS-PAGE gel and transferred onto polyvinylidenedifluoride (PVDF) membrane. After blocking in BSA 5% TBS-Tween 20 0.1%, membranes were then probed with appropriate antibodies (dilution 1/1000) to assess total protein level. The appropriate secondary antibody conjugated to HRP and the BM chemiluminescence blotting system (Roche Molecular Systems, Bale, Switzerland) were used for detection. Antibodies used for these experiments are as follows: OGT (Cell Signalling Technology Inc. Danvers, Massachusetts, United States), OGA (Santa Cruz Biotechnology, Dallas, Texas, United States), Alpha tubulin (ThermoFisher Scientific Inc., Waltham, Massachusetts, United States), TOM20 (ThermoFisher), Histone 3 (Cell Signaling), OXPHOS (Abcam), NDUFS1 (Proteintech, Rosemont, Illinois, United States) and O-GlcNAc-HRP (Abcam). The uncropped version of all the blots presented in main figures can be found in Supplementary Figures S5 and 6.

For the immunoblotting of specific mitochondrial proteins after O-GlcNAc immunoprecipitation, 250 µg (isolated mitochondria) or 500 µg (total heart homogenate) of protein samples were immunoprecipitated with anti-O-GlcNAc RL2 antibody (1 μg, Abcam) overnight at 4 °C following pre-clearing of the lysate with pre-washed protein G Sepharose beads. After three washing with TBS (50 mM Tris-Cl, 150 mM NaCl at pH 7.6), immunoprecipitated O-GlcNAc proteins were eluted with Laemmli buffer and boiled 10 min at 100 °C. Protein separation was performed by SDS-PAGE and immunoblotting was realised as mentioned above with NDUFS1 or OXPHOS antibodies.

### Mass spectrometry and protein identification

#### Preparation of proteins

Isolated mitochondria pellets or 20 mg of freeze-clamped hearts were homogenized in 200 µL of RIPA lysis buffer (25 mM Tris HCl, 150 mM NaCl, 1% NP-40, 1% sodium deoxycholate, 0.1% SDS at pH 7.6) supplemented with a protease/phosphatase inhibitor cocktail (ThermoFisher) and 1 µM of O-GlcNAc cycling enzyme inhibitors (Sigma-Aldrich, Saint-Louis, Missouri, United-States). 250 µg of proteins from the lysate were then precipitated using chloroform/methanol (MeOH) precipitation method as follows. 600 μL of MeOH were added to the 200 μL sample, followed by 150 μL of chloroform and 400 μL of 18 megaOhm water. Samples were then vortexed briefly and centrifuged for 5 min at 13,000×*g*. The upper aqueous phase was carefully removed and discarded. Additional 450 μL MeOH were added to pellet the protein after brief vortex and centrifugeation for 5 min at 13,000×*g*. The supernatant was then removed, and the pellet air dried for 5 min. Finally, proteins were resuspended in 40 µL of 1% SDS in 20 mM HEPES pH 7.9 and heated 5-10 min at 90 °C to assure completely resuspension of proteins.

#### Enzymatic labelling and O-GlcNAcylated protein enrichment

O-GlcNAc groups from proteins were labelled with tetramethylrhodamine azide (TAMRA) using the Click-iT® O-GlcNAc enzymatic labelling system kit (C33368) followed by the Click-iT® protein analysis detection kit (C33370) from Invitrogen according to the manufacturer’s instructions. SDS was then quenched with NEFTD buffer (100 mM NaCl, 50 mMTris-HCl, 5 mM EDTA, 6% NP-40 at pH 7.4). Before immunoprecipitation of TAMRA labelled proteins, lysate was precleared with washed protein G sepharose beads to avoid non-specific binding of proteins on the beads. Afterwards, supernatant was incubated with pre-washed protein G sepharose beads (10 µL) coupled with anti-TAMRA antibody (10 µg, A6397, Invitrogen) for 1.5 h at 4 °C. Following centrifugation (500×*g*, 1 min), the beads were washed once with NEFTD buffer (100 mM NaCl, 50 mM Tris-HCl pH 7.4, 5 mM EDTA, 6% NP-40) and three times with NEFT buffer (NEFTD without NP-40). The beads were then boiled 5 min in Laemmli buffer (2 mM EDTA, 4% SDS, 20% Glycerol, 0.004% bromophenol blue, 50 mM DTT, and 100 mM Tris at pH 6.8) to elute O-GlcNAc proteins. Proteins were then separated on 1 mm on SDS-PAGE gel and stained with Coomassie blue (Sigma-Aldrich).

#### In-Gel Digestion and identification of captured O-GlcNAc Proteins

Gels were cut in seven bands of equal size and in-gel digested with trypsin. Peptides separation was performed using a C18 reversed-phase analytical column (Thermo Scientific) on an Ultimate 3000-nLC RSLC system. The peptides were subjected to Nano-Spray-Ionization source followed by tandem MS/MS in a tribrid Fusion Lumos Orbitap analyser coupled online to the nano-LC. Spectra were acquired by a data dependent scan routine with ion precursor detection in the Orbitrap and daughter ions in the Iontrap. The resulting MS/MS data were processed using Sequest HT search engine within Proteome Discoverer 2.4 against a rat protein database obtained from Uniprot (29 953 entries). Trypsin was specified as cleavage enzyme allowing up to 2 missed cleavages, 4 modifications per peptide and up to 5 charges. Mass error was set to 10 ppm for precursor ions and 0.1 Da for fragment ions. Oxidation on methionine, carbamidomethyl on cysteine were considered as variable modifications. False discovery rate (FDR) was assessed using Percolator and thresholds for protein, peptide and modification site were specified at 1%. The filtered Sequest HT output files for each peptide were grouped according to the protein from which they were derived and abundance was evaluated by label-free quantification within Proteome Discoverer from area under the curve of MS1 intensities. Following such procedure, we were able to identify 2534 putative O-GlcNAcylated proteins.

#### LC-MS/MS data analysis

The analysis was conducted in R (R Core Team, 2020). The expression values were log2 transformed but not subjected to normalization given the data acquisition technique involving an enrichment of O-GlcNAc proteins through immunoprecipitation prior to MS analysis. To maximize stringency, only proteins reliably detected in all experimental replicates (*n* = 4) from control and NButGT-treated mitochondria were considered. Of the 842 proteins identified, mitochondrial proteins (409) were selected according to their mitochondrial status from the Mitominer database. Prior to differential expression analysis and based on exploratory analysis, NButGT-treated replicate n°3 was removed as it did not respond to treatment (assessed by O-GlcNAc immunoblotting). The differential expression between control and NButGT-treated groups was statistically assessed through linear models using empirical Bayes methods for variance modelling, as implemented in the R/Bioconductor limma package^42,43^ where the group effect was the only one included in the modes. P-values for the group effect were adjusted for multiple testing with the Benjamini-Hochberg FDR procedure. Proteins having a group effect with an adjusted *p*-value (*q*) < 0.1 and *q* < 0.05 were arbitrary considered differentially expressed between control and NButGT-treated mitochondria with either high or very high confidence score, respectively.

### Pathway analysis and bioinformatics

To identify the pathway annotations of the mitochondrial O-GlcNAcylome, the list of proteins displaying significantly increased O-GlcNAcylation was ranked according to q values and uploaded into the g:Profiler^44^ platform, and an ordered query was performed using the *Rattus norvegicus* database. KEGG (metabolism, cellular processes, human diseases) and REACTOME (biochemical pathways) terms annotating 350 proteins or less were considered in order to filter out large annotations that provide limited interpretative value^45^. The g:SCS (shortest common superstring) algorithm was used for multiple hypothesis testing corrections using a default *alpha* threshold of 0.05^44^. The ggplot2 R package was used to generate bubble plots in which pathway enrichments were expressed as Rich Factors, which represents the ratio of the number of proteins observed for a given pathway term to the total number of proteins for this term. The Cytoscape (version 3.8.0) stringApp plugin was used to import protein-protein interaction data from STRING^46^. For this analysis, an interaction score of 0.7 (high confidence based on default active interaction sources) was set as minimum. Clustering of the STRING network was performed with the Markov Cluster (MCL) algorithm with a granular parameter set at 4. The Auto-annotate plugin^47^ of Cytoscape was used to identify pathways/processes corresponding to these protein clusters based on Stringdb description and GO annotations. To compare overlap between distinct O-GlcNAcylomic datasets, Venn diagrams were generated using the Eulerr R Package. Hypergeometric tests were used to determine the statistical significance of the actual *vs* expected number of shared hyper-O-GlcNAcylated proteins between datasets.

### Statistics and reproducibility

For functional analyses, values are reported as mean ± sem for a minimum of 3 biological replicates, with 2-3 experimental replicates per experimental groups. Data are graphically represented as histograms. Paired two-sided *t-tests* (GraphPad Prism 8.4.3) were used to determine statistical difference when two means were compared, with a significance threshold set at *p* < 0.05.

For proteomics, values reported were obtained from 3-4 biological replicates. Data are depicted as volcano plots or histograms of mean Log2 FC ± sem with values for individual proteins represented by dots. Difference in the abundance of individual proteins between groups was determined by linear models using empirical Bayes methods for variance modelling. For each *p* value obtained, a corresponding FDR was calculated according to the Benjamini and Hochberg method. Proteins having an adjusted *p*-value (*q*) < 0.1 and *q* < 0.05 were arbitrary considered differentially expressed between control and NButGT-treated mitochondria with either high or very high confidence score, respectively (Supplementary Table 1).

### Reporting summary

Further information on research design is available in the Nature Research Reporting Summary linked to this article.

## Supplementary information

Supplementary Information

Description of Additional Supplementary Files

Supplementary Data 1

Reporting Summary

## Data Availability

The mass spectrometry proteomics data have been deposited to the ProteomeXchange Consortium via the PRIDE partner repository with the dataset identifier PXD026495. Source data for the graphs and charts is available as Supplementary Data 1 and any remaining information can be obtained from the corresponding author upon reasonable request: Yan Burelle (yburell2@uottawa.ca) and Luc Bertrand (luc.bertrand@uclouvain.be). All requests will need to specify how the data will be used and will require approval by co-investigators.
