# Supplementary material for: The intra-mitochondrial O-GlcNAcylation system rapidly modulates OXPHOS function and ROS release in the heart
Source: Commun Biol. 2022 Apr 12;5:349. doi: 10.1038/s42003-022-03282-3 (PMC9005719; doi:10.1038/s42003-022-03282-3)
Supplement: Supplementary file 1 — Supplementary Information [file 42003_2022_3282_MOESM1_ESM.pdf]

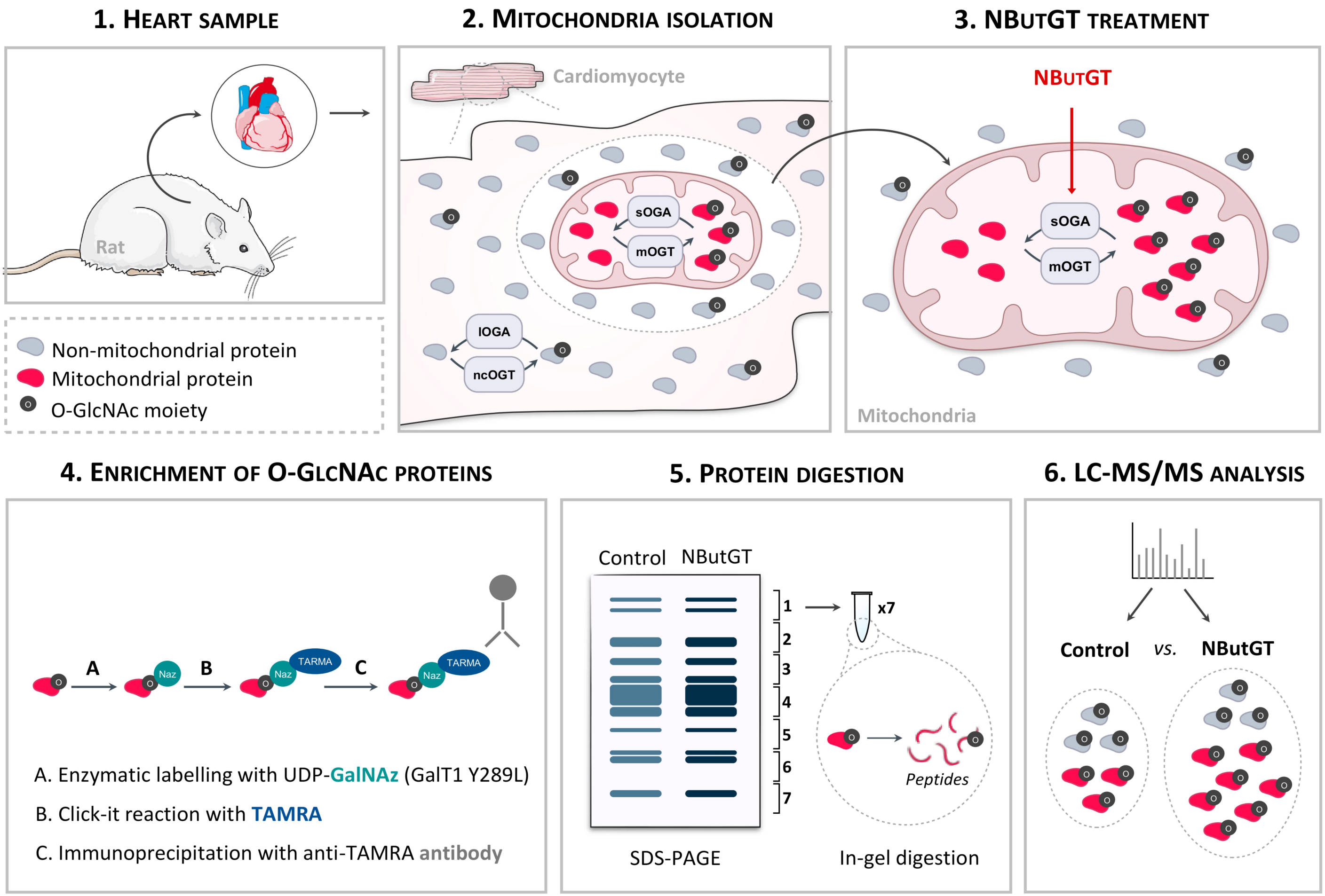

Supplementary Figure S1: Schematic overview of the experimental approach used to investigate the intra-mitochondrial O-GlcNAcylation system. (1) Crude mitochondrial fractions were isolated from rat hearts using a standard differential centrifugation protocol. (2) This procedure allowed to obtained preparations enriched with mitochondria, along with some typical protein contaminants arising from other cellular fractions, which included trace levels of nucleocytoplasmic OGT and OGA isoforms (Fig 1a-b). (3) Mitochondria were incubated in vitro during 30 min in presence of UDP-GlcNAc and NButGT or their vehicle (H2O). Following lysis of mitochondrial pellets and protein extraction, equal amounts of proteins were submitted to enzymatic labeling of O-GlcNAc moieties and subsequent immunoprecipitation with an anti-TAMRA antibody. (5) Immuno-precipitates were resolved by SDS-PAGE and submitted to in-gel trypsin digestion. (6) Equal volumes of tryptic digests containing variable amounts of O-GlcNAcylated peptides were submitted to LC-MS/MS analysis. Identified peptides were grouped according to the protein from which they were derived and abundance was quantified from area under the curve of MS1 intensities. Protein subcellular localizations were determinate by the Mitominer Data base and final data were log two transformed and expressed as FC vs control.

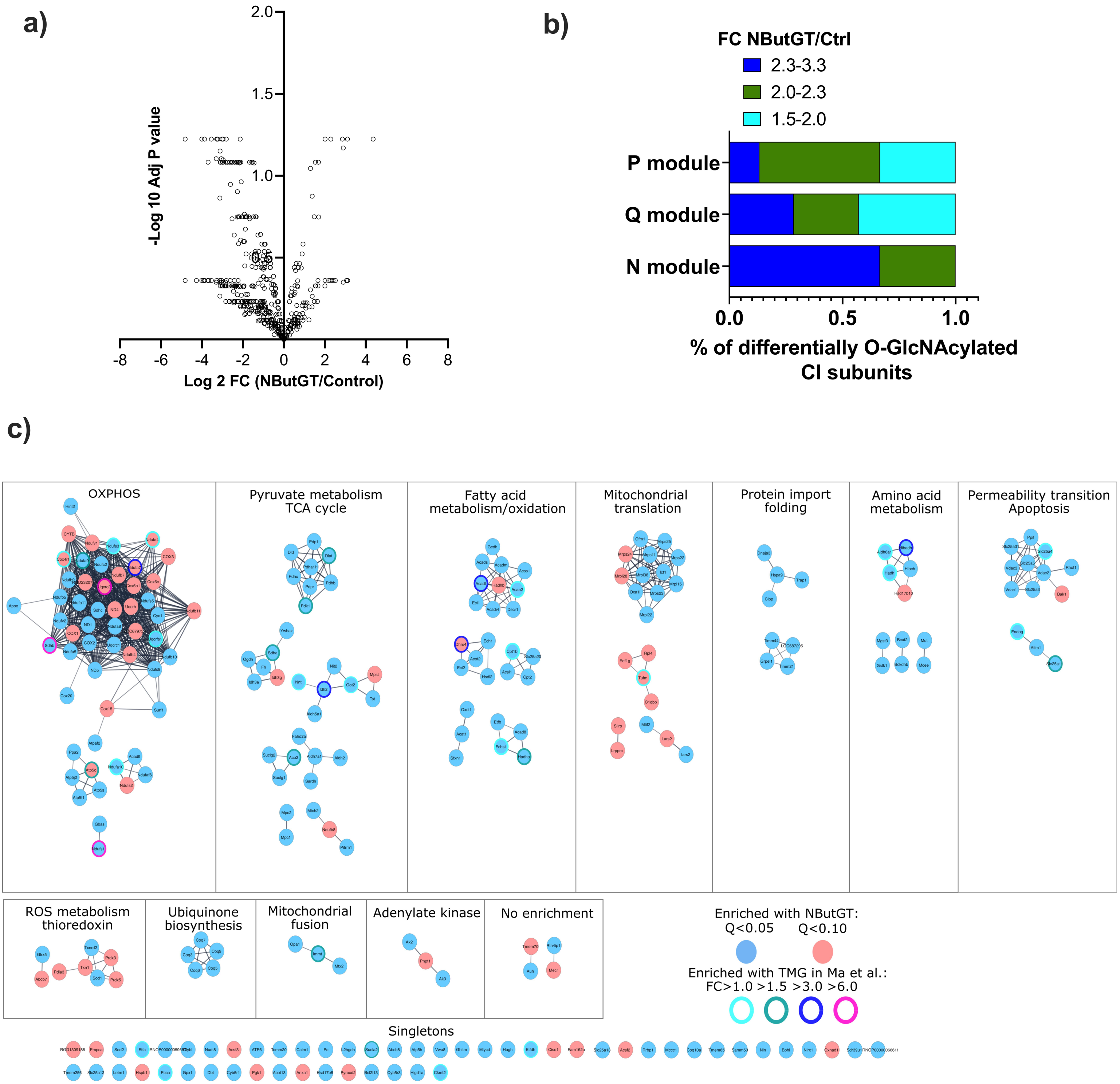

Supplementary Figure S2: Investigation of non-mitochondrial and mitochondrial protein O-GlcNAcylation following OGA inhibition. a) Volcano plot analysis showing the impact of NButGT O-GlcNAcylation for proteins with a non-mitochondrial status on the Mitominer database. Statistical analysis was assessed using a linear regression model (empirical Bayes methods) followed by the Benjamini-Hochberg FDR procedure**. b)** Fold change in the abundance of O-GlcNAc-modified proteins between control and NButGT-treated mitochondria according to their localization in the different nodules of the Complex I. **c)** STRING network of mitochondrial proteins displaying increased O-GlcNAcylation in response to NButGT in the present study and in the study by Ma *et al* [20]. Clustering was performed with the Markov Cluster (MCL) algorithm with a granular parameter set at 4. The Auto-annotate function of Cytoscape was used to identify pathways/processes corresponding to these clusters based on Stringdb description and GO annotations. Nodes were color coded according to the q values observed in the present study, while borders were color coded assorting to FC in O-GlcNAcylation observed in the Ma *et al*. study.

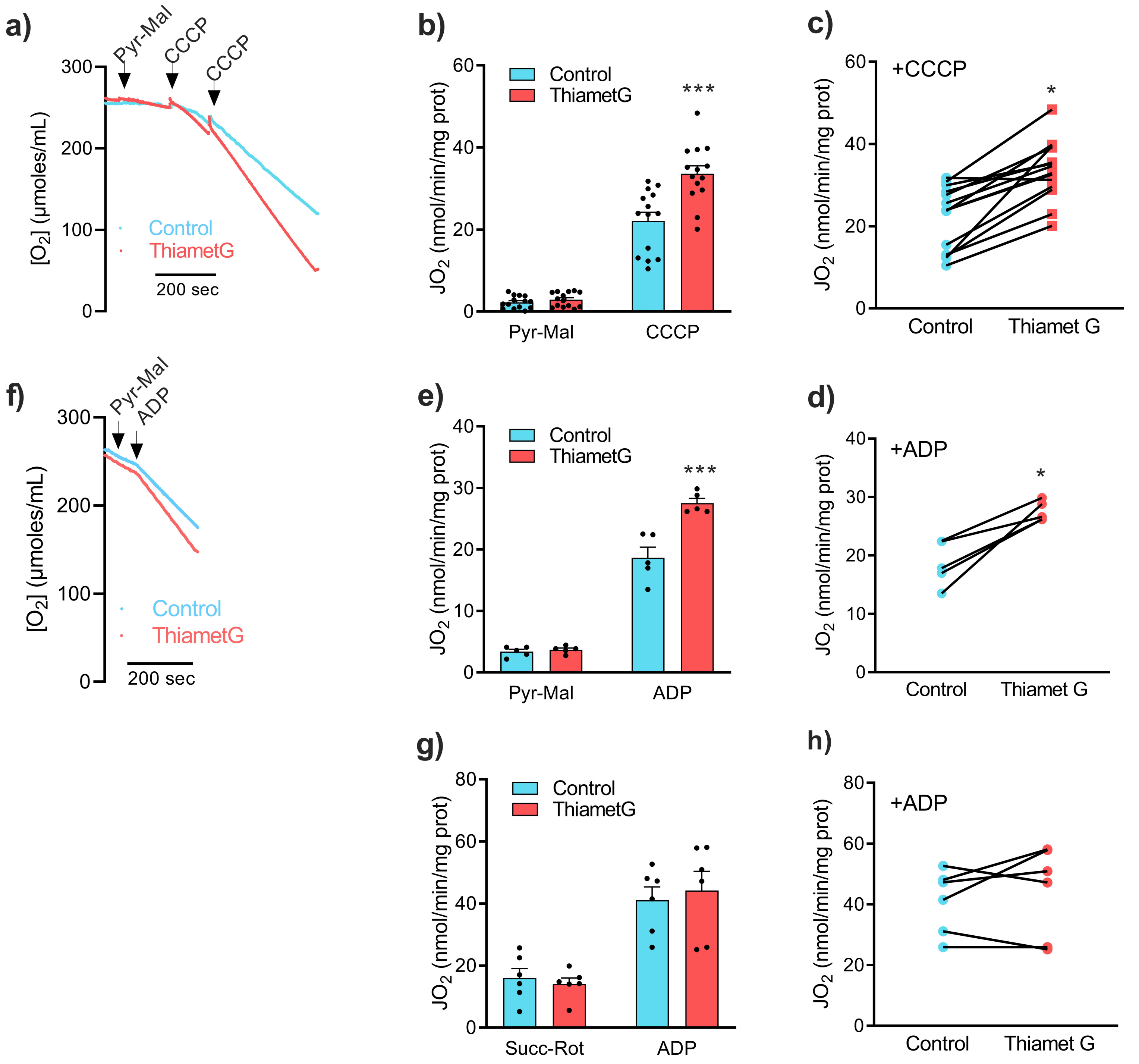

Supplementary Figure S3: Impact of Thiamet G-induced mitochondrial O-GlcNAcylation on respiratory function. Following pre-incubation with UDP-GlcNAc in absence or presence of Thiamet G (5 µM), mitochondria were transferred to respirometry chambers for the recording of baseline state 2, maximal ADP-stimulated (ADP), and CCCP uncoupled respiration in presence of complex I (Pyruvate-Malate [Pyr-Mal]) or complex II (Succinate in presence of the complex I inhibitor rotenone [Succ-Rot]) substrates. For all experiments, control and Thiamet G-treated mitochondria were tested in parallel, allowing pairwise comparisons. Panels **a** and **d** show representative respirometry tracings. Panels **b**, **e** and **g** show the calculated means ± sem for each respiratory state in the two experimental groups. Panels **c**, **f** and **h** illustrate the effect of Thiamet G on ADP stimulated (F-H) or CCCP uncoupled respiration (H) for each of the paired incubations (3 biological replicates with 1-3 technical replicates per group).

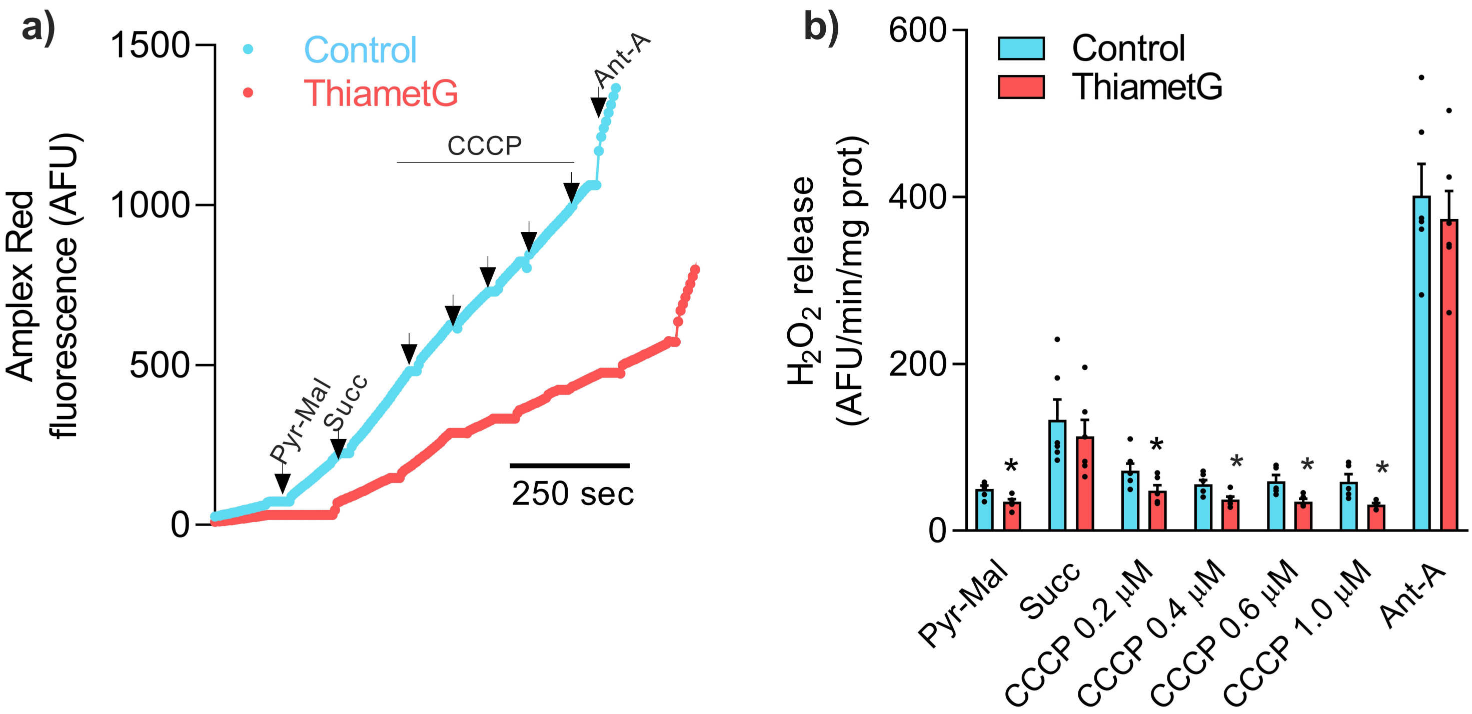

Supplementary Figure S3: Impact of Thiamet G-induced mitochondrial O-GlcNAcylation on respiratory function. Following pre-incubation with UDP-GlcNAc in absence or presence of Thiamet G (5 µM), mitochondria were transferred to respirometry chambers for the recording of baseline state 2, maximal ADP-stimulated (ADP), and CCCP uncoupled respiration in presence of complex I (Pyruvate-Malate [Pyr-Mal]) or complex II (Succinate in presence of the complex I inhibitor rotenone [Succ-Rot]) substrates. For all experiments, control and Thiamet G-treated mitochondria were tested in parallel, allowing pairwise comparisons. Panels **a** and **d** show representative respirometry tracings. Panels **b**, **e** and **g** show the calculated means ± sem for each respiratory state in the two experimental groups. Panels **c**, **f** and **h** illustrate the effect of Thiamet G on ADP stimulated (F-H) or CCCP uncoupled respiration (H) for each of the paired incubations (3 biological replicates with 1-3 technical replicates per group).

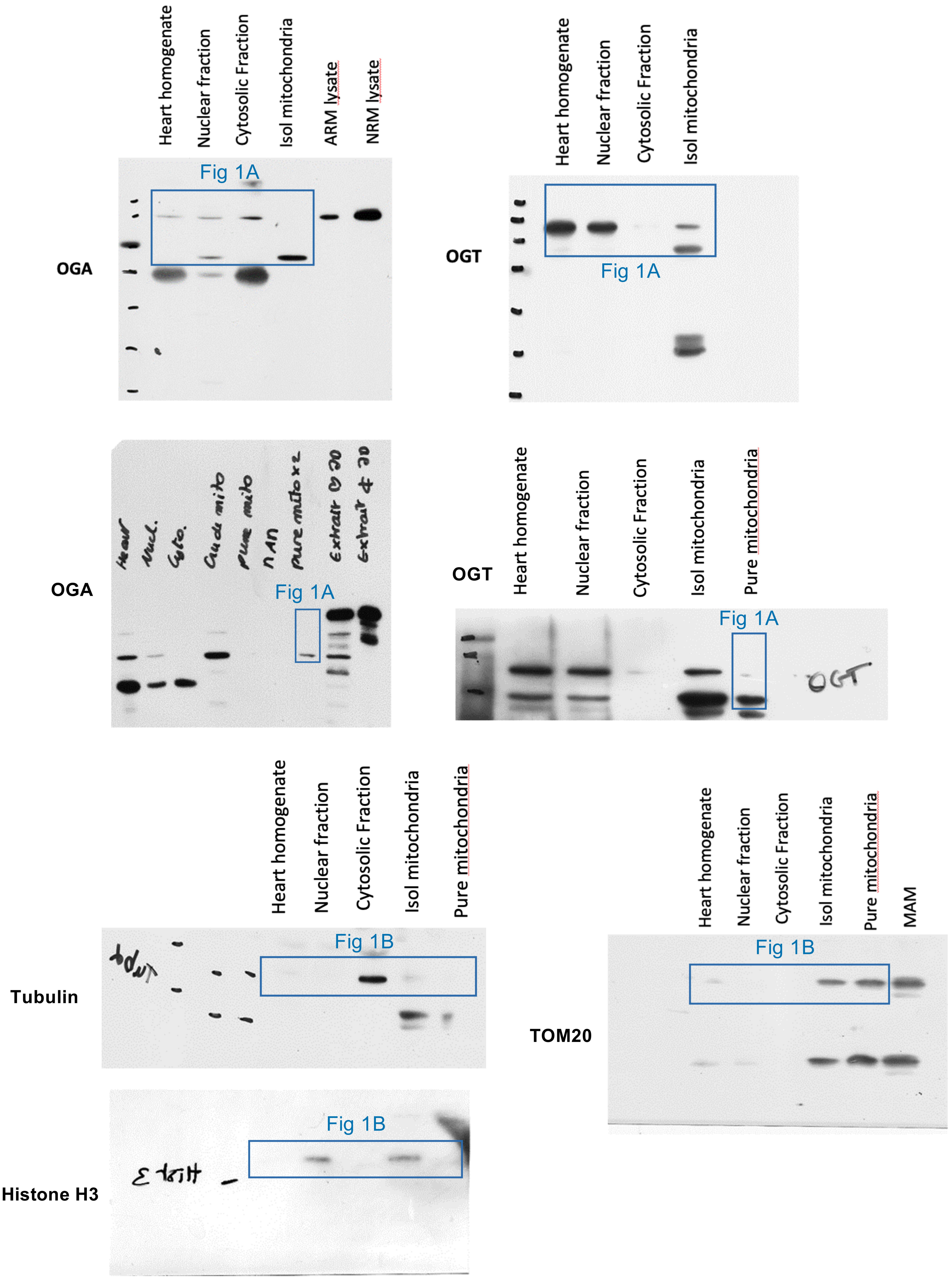

Supplementary Figure S5: Unprocessed images of membranes used for immunoblots presented in Fig 1.

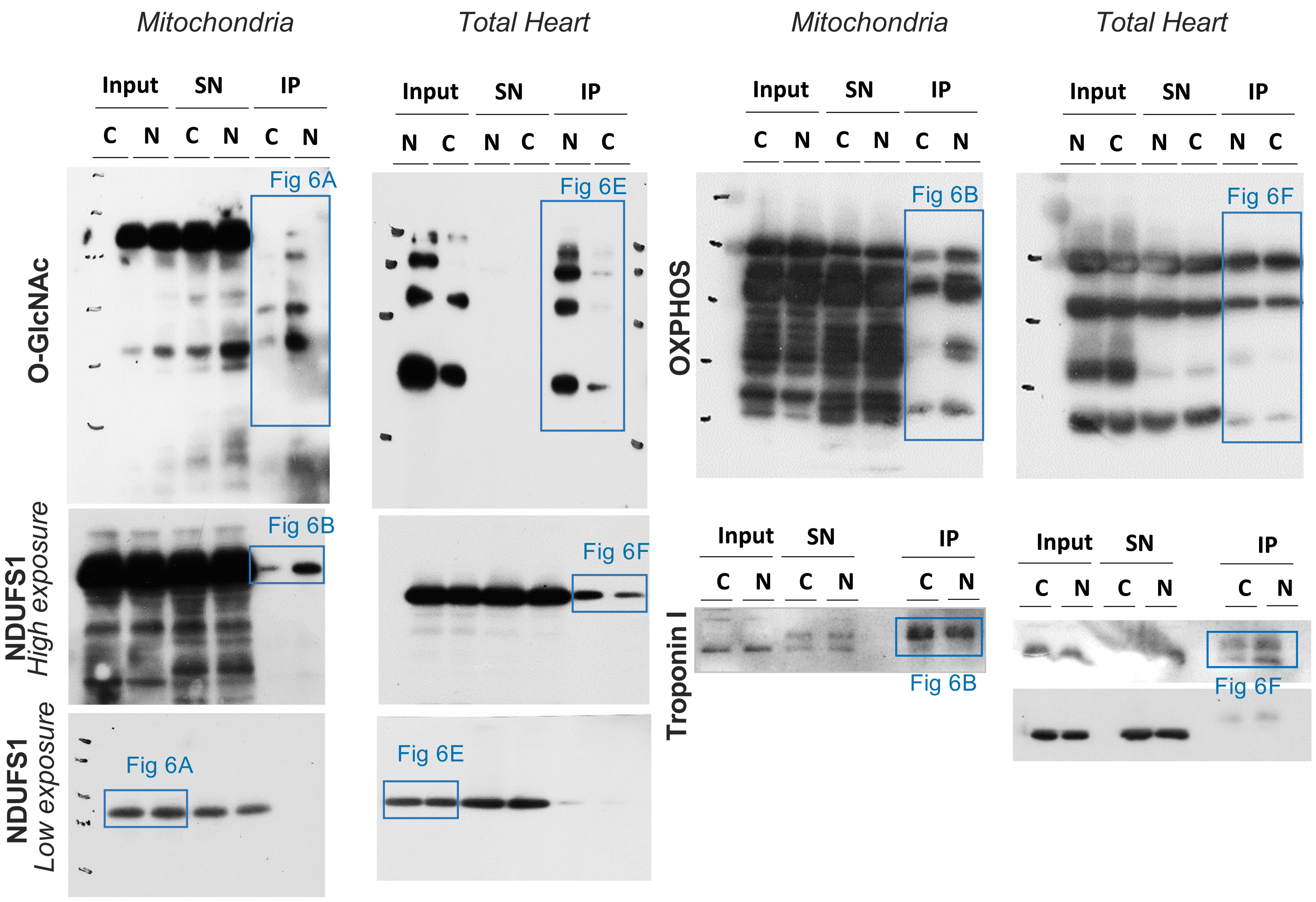

Supplementary Figure S6: Unprocessed images of membranes used for immunoblots presented in Fig 6A-F.

Supplementary Table S1: Comparative analysis of O-GlcNAcylated proteins following in vitro or in vivo NButGT treatment. List of O-GlcNAcylated proteins identified obtained following in vitro exposure of isolated mitochondria to NButGT (#1) compared to cardiac O-glcNAcylomic dataset derived from mice treated with NButGT (#2). Dark blue cells highlight proteins with FC>1,5 (Log2FC>0,58); medium FC>1,2 (Log2FC>0,26) and light blue FC>1 (Log2FC>0).

#1 (in vitro)			#2 (in vivo)
**Gene**	**AdjPval**	**Log2FC**	**Log2FC**
Mlycd	0,036219	5,0542383	-0,22302367
Mrpl38	0,037828	4,5266909	-0,05239726
Tst	0,037828	4,4831217	-0,48521952
Gpx1	0,037828	4,3583643	0,03593602
Rtn4ip1	0,037828	4,3249218	0,51133591
Abcb8	0,037828	4,3111377	0,01807561
Nudt8	0,037828	4,1995844	-0,29416556
Ndufaf6	0,036219	4,1436254	0,31476548
Clybl	0,037828	3,9171971	0,00199168
Vwa8	0,037828	3,9128006	1,11277804
Mrpl15	0,036219	3,9010537	0,83132887
Fahd2a	0,036219	3,8808771	NA
Cyb5r3	0,037828	3,7295167	0,27592267
Oxa1l	0,037828	3,6297092	0,38995933
Clpp	0,037828	3,597414	0,14744399
Kat3	0,037828	3,4817683	NA
Nlrx1	0,037828	3,4501217	0,41551219
Rpl4	0,077144	3,3302439	-0,13896196
Higd1a	0,037828	3,3194459	1,06498434
Tmem65	0,037828	3,316931	0,53950296
Nit2	0,037828	3,3030395	0,03877761
Grpel1	0,037828	3,2851366	0,03618385
Timm50	0,037828	3,2645057	0,19863208
Letm1	0,037828	3,2629061	0,59281113
Aldh7a1	0,037828	3,2553931	NA
Ndufa8	0,041421	3,2462646	0,31652351
Lars2	0,054815	3,2389534	0,28823996
Auh	0,037828	3,2363892	0,62162374
Ppa2	0,037828	3,2139048	-0,01256295
Timm44	0,037828	3,2081809	0,0156414
Coq7	0,037828	3,1155229	0,05132324
Gstk1	0,037828	3,1023771	-0,13063292
Hsd17b8	0,037828	3,0912617	-0,09032497
Pitrm1	0,037828	3,0655927	1,15560495
Sardh	0,037828	3,058349	NA
Aldh5a1	0,038248	3,0179449	0,19133749
L2hgdh	0,037828	3,009186	0,17858421
Mrps23	0,037828	2,9945447	0,25893335
Endog	0,037828	2,981828	-0,03212739
Mut	0,037828	2,976578	NA
Atp5s	0,037828	2,9663696	NA
Acad8	0,037828	2,9357018	-0,1873788
Eci1	0,037828	2,9244637	0,17686736
Fh	0,037828	2,9209375	0,46170735
Slc25a4	0,037828	2,911997	0,27092103
Pdp1	0,049901	2,9056392	0,05172957
Pc	0,037828	2,8987469	NA
Iars2	0,037828	2,8971452	0,06725274
Mecr	0,050603	2,8961787	-0,13667286
Etfa	0,037828	2,8958899	0,12607806
Hadh	0,037828	2,8954573	0,3014456
Atp5f1	0,037828	2,8937595	0,22458568
Cox20	0,037828	2,8880063	-0,47764041
Pdk1	0,037828	2,884855	0,39079188
Acad9	0,037828	2,8827262	0,41086873
Atpaf2	0,037828	2,8768181	-0,36720423
Mpc1	0,037828	2,8688056	0,01603131
Slc25a3	0,037828	2,8623411	0,63611025
Acadvl	0,037828	2,854331	0,47709402
Ndufv2	0,037828	2,8420339	-0,17590218
Samm50	0,038248	2,8376604	0,35005203
Hagh	0,039989	2,8347035	0,13422073
Acot2	0,037828	2,8298966	0,23839452
Echs1	0,037828	2,8266167	0,10699377

Coq6	0,038248	2,8187113	0,1784542	Mccc1	0,037828	2,6400616	0,25181383
Mtch2	0,037828	2,808325	0,31751727	Cpt2	0,037828	2,633844	0,18325402
Immt	0,037828	2,7990808	0,58477071	Aldh6a1	0,037828	2,6282189	0,32464817
Mrps11	0,037828	2,7918282	NA	Acss1	0,037828	2,6274513	0,29624419
Apoo	0,037828	2,7886388	0,14772638	Suclg2	0,047975	2,6255502	0,09860447
Ech1	0,037828	2,7816691	0,10129361	Rhot1	0,037828	2,6178301	1,13652091
Opa1	0,037828	2,7675834	0,1445723	Ndufs8	0,037828	2,6154271	-0,18259464
Ghitm	0,037828	2,7654367	-1,60828548	Sfxn1	0,037828	2,6077182	NA
Nnt	0,037828	2,7628583	0,24674829	Hadha	0,037828	2,5933258	0,35682994
Hibch	0,037828	2,7505282	0,11236017	Decr1	0,037828	2,5900095	0,15330168
Mpc2	0,037828	2,7376082	0,63947703	Cpt1b	0,037828	2,5895803	0,29122348
Acsl1	0,037828	2,7342343	0,38325043	Dbt	0,041732	2,5887541	0,16063568
Coq9	0,037828	2,7290267	0,45296988	Coq3	0,037828	2,5883947	0,37208171
Etfb	0,037828	2,7286495	-0,02287712	Idh3a	0,038248	2,5876519	-0,07228971
Bckdhb	0,037828	2,7274874	0,21908694	Cyb5r1	0,041732	2,587069	-0,17730635
Pdpr	0,037828	2,7263795	-0,85281616	Hk1	0,037828	2,5834625	0,21251284
Ndufs1	0,037828	2,7218487	0,89224158	Hsdl2	0,037828	2,5756094	0,3522163
Slc25a11	0,037828	2,7179829	0,52532758	Hspa9	0,037828	2,5718361	0,40142143
Trap1	0,037828	2,715586	0,45402647	Ak2	0,038695	2,5661968	-0,11756827
Ak3	0,041732	2,7147469	-0,15852255	Ckmt2	0,040097	2,5467931	0,18855786
Pdhb	0,037828	2,7054367	0,20862285	Mgst3	0,037828	2,5442059	0,30337081
Bcl2l13	0,037828	2,6962728	0,33733687	Coq5	0,037828	2,5427428	-0,02768279
Ppif	0,037828	2,6911446	0,1198819	Slc25a31	0,047975	2,5333906	0,28761551
Surf1	0,037828	2,6901814	NA	Cyc1	0,037828	2,5197744	0,26347057
Hibadh	0,037828	2,6882363	-0,05041585	Pcca	0,037828	2,5150739	0,66340991
Txnrd2	0,038248	2,6870513	-0,2355221	Gfm1	0,037828	2,5105962	0,40625412
Slc25a5	0,037828	2,6823906	0,31639927	Mcee	0,037828	2,5099253	0,83080365
Mtif2	0,041732	2,6815279	-0,21269366	Gcdh	0,041732	2,509225	0,45885963
Sod2	0,037828	2,6749182	0,00976056	Etfdh	0,037828	2,4974547	0,58959335
Bcat2	0,038248	2,6568079	0,00804277	Aifm1	0,037828	2,4850375	0,93064635
Bphl	0,041732	2,6560808	-0,09641509	Got2	0,039598	2,4801307	0,22565213
Mrps22	0,044297	2,6519941	0,32721033	Atp5h	0,041732	2,4676246	-0,13152819
Nln	0,037828	2,6501502	-0,16074895	Gatd3a	0,037828	2,46485	NA

Mtx2	0,041732	2,4635693	0,051137	Ndufb9	0,038695	2,2803266	0,18187443
Pdhx	0,037828	2,4557953	0,49045684	Timm29	0,054815	2,2728878	-0,55238273
Sdhc	0,041732	2,4545357	0,02139544	Ndufa11	0,041732	2,2639729	0,55154816
Dnaja3	0,04552	2,4460635	0,0697668	Eci2	0,049843	2,2639217	0,35790967
Mrps25	0,037828	2,4458793	NA	Uqcrh	0,081746	2,2626072	NA
Uqcrc1	0,041104	2,4435604	0,10731751	Aldh2	0,041732	2,2595511	0,04998952
Aco2	0,037828	2,4389872	0,26036869	Ndufb5	0,039989	2,2582696	0,54116387
Cox6c	0,054815	2,4350818	-0,16707584	Mrpl28	0,05463	2,2562575	0,69232263
Pdha1l1	0,041104	2,4229818	0,23488777	Acadm	0,042717	2,2500718	0,18328941
Idh3g	0,044297	2,4148195	0,30119423	Atp5j2	0,041623	2,2482512	0,58941599
Sdhb	0,038248	2,4077221	-0,15465072	Mpst	0,05802	2,2356737	1,48322926
Acaa2	0,041732	2,4061614	0,1056513	Sdha	0,041732	2,2226005	0,30111375
Ogdh	0,037828	2,4038714	0,24839054	Mrpl22	0,041732	2,2209564	0,60004664
Oxct1	0,041104	2,4002236	0,29295506	Sdr39u1	0,041732	2,2130011	-0,07179587
Slc25a12	0,038248	2,3888083	0,07517014	Suclg1	0,044297	2,2122551	0,25203841
Mrpl58	0,039598	2,3718849	NA	Slc25a20	0,041732	2,2110913	0,29244073
Sucla2	0,041732	2,366532	0,0643007	Atp5me	0,048428	2,2096749	1,03973947
Slc25a13	0,041732	2,3651175	0,14124044	Ndufb10	0,044297	2,2048793	0,10287517
Dlat	0,037828	2,3631739	0,29052914	Mtnd1	0,041932	2,186787	0,90557082
Cox15	0,093831	2,3493059	-0,23037647	Mtco2	0,041732	2,1852467	-0,26468431
Tomm20	0,04102	2,3486184	0,65501152	Dhrs4	0,056845	2,1707468	-0,00812237
Uqcrfs1	0,041732	2,3449079	0,21166798	Vdac2	0,041732	2,1500778	1,08210663
Acat1	0,041732	2,3197278	0,12786179	ATP6	0,044297	2,1484145	0,40081305
Idh2	0,041732	2,3187883	0,24986006	Acot13	0,040769	2,13823	0,27044663
Ndufc2	0,037828	2,304323	0,85115054	Tmem256	0,040014	2,1350548	-0,24058718
Glrx5	0,041732	2,3038867	NA	Ndufv1	0,053886	2,1339514	0,24384278
Ndufa5	0,041732	2,301498	0,23481069	Hint2	0,041732	2,1314132	0,80723543
Ndufa10	0,042443	2,3014062	0,27033212	Prdx3	0,056845	2,1238232	-0,05107579
Atp5f1a	0,042265	2,2911764	0,4171115	Tufm	0,061548	2,1150426	0,11488558
Dld	0,041732	2,28971	0,4167506	Ndufs2	0,05463	2,111338	0,21991198
Acads	0,041732	2,2848622	0,16549508	C1qbp	0,077144	2,1052803	-0,10425796
Vdac1	0,040699	2,2845899	0,0024684	Ndufs3	0,047975	2,1002756	-0,01965294
Pmpca	0,071467	2,2807939	0,44809247	Acadl	0,049879	2,0994742	0,23963326

Nipsnap2	0,048428	2,0838596	-0,08071942
Vdac3	0,049901	2,0776962	0,28015942
Mtnd5	0,049843	2,0670821	0,05824992
Ndufs5	0,042398	2,0586188	0,62519793
Ndufa9	0,049706	2,0585522	1,26823594
Pyroxd2	0,067373	2,0555037	NA
Uqcrc2	0,059171	2,047706	0,00274392
Hadhb	0,056845	2,044118	-0,01067902
Slirp	0,051908	2,0054882	NA
Ndufb11	0,05728	2,0046953	0,43682147
Ndufb7	0,054815	1,9999401	1,46251693
Acsf3	0,080354	1,9914499	0,47113302
Coq10a	0,048428	1,9685649	0,02207303
Rrbp1	0,049901	1,9646757	0,22766867
Mrps24	0,053115	1,9614362	0,09247896
Ak4	0,169745	1,9540206	1,18006167
Abcb7	0,062066	1,9527762	-0,12456679
Timm21	0,049901	1,9515541	NA
Cisd1	0,057276	1,9496498	1,3892039
Lrpprc	0,073798	1,9429428	0,25534361
Hsd17b10	0,073798	1,9359112	0,04472029
Acsf2	0,059171	1,9309549	0,10074094
Atp5o	0,053101	1,9129802	-0,19233187
Pnpt1	0,074926	1,8902753	-0,20168422
Mtnd4	0,061548	1,8843288	0,35727065
Mt-Cyb	0,061054	1,8666031	NA
Uqcr10	0,089656	1,8445038	0,46524642
Ndufa7	0,061548	1,8271921	0,71831609
Ptges2	0,375967	1,8127957	-0,63314425
Cox4i1	0,079529	1,8084924	0,38431826
Prdx5	0,064383	1,8014474	0,51658331
Acad10	0,184539	1,7760286	NA
Pdk2	0,169449	1,7711309	0,51844296
Mtco3	0,074822	1,7641934	-0,11929586
Naxe	0,172205	1,7241928	NA
Tmem70	0,079219	1,7209715	NA
Cox6b1	0,077144	1,7202419	-1,88268291
Fam162a	0,074762	1,7107557	0,33906577
Coq8a	0,141677	1,6941973	0,60963251
LOC100912599	0,079529	1,6905654	NA
Ndufa4	0,093831	1,6877707	0,60129457
Mtco1	0,093831	1,676077	NA
Ndufb4	0,081552	1,6615315	0,05989266
Atp5f1b	0,081213	1,6594224	0,69495158
Idh3B	0,123628	1,6590926	0,23812006
Ndufab1	0,115825	1,6482976	0,46687922
Bckdha	0,13573	1,6409483	0,42210959
Apool	0,128537	1,6244624	0,3641231
Oxnad1	0,075663	1,6223195	-0,04738238
Ndufb8	0,096144	1,6084916	0,33514254
Mdh2	0,122126	1,581564	0,03562464
Slc25a42	0,21108	1,5711377	0,25243384
Gfm2	0,106798	1,546498	0,190473
Crat	0,113961	1,5309961	0,24179731
Mrpl21	0,11652	1,5202367	0,38833153
Dlst	0,123628	1,5160175	0,29093977
Mrpl27	0,119674	1,4872799	NA
Atp5f1e	0,120837	1,477325	0,37386784
Phb	0,153635	1,4730678	-0,25705859
Cox5b	0,150076	1,469482	0,26995404
Ndufaf3	0,131832	1,4670782	0,64708666
Ndufa6	0,141161	1,4637284	0,5347326
Aldh9a1	0,208633	1,4617295	0,39107334
Cox7a2l	0,153957	1,4270863	NA

Ndufa2	0,118982	1,4259823	-0,29656537
Me3	0,303356	1,4218338	0,28866087
Tbrg4	0,246897	1,4187769	1,07749441
Rps14	0,230567	1,414642	NA
Glud1	0,204253	1,4061199	1,39987719
Ndufs7	0,204253	1,3877684	0,81023037
Timm22	0,142482	1,3845505	-0,35411841
Atp5l	0,172205	1,3694716	0,42857388
Pdf	0,138864	1,3649293	NA
Afg3l2	0,1673	1,3466654	0,03669781
Cmc1	0,280763	1,3442023	NA
Ivd	0,174029	1,3092634	-0,06561046
Isca2	0,169449	1,3065557	NA
Phb2	0,254371	1,2737454	0,27043876
Slc27a1	0,179403	1,2641414	0,83421505
Cox5a	0,192714	1,2611296	0,37937444
Cs	0,243672	1,1500017	0,13066695
Ndufa13	0,281546	1,1097151	0,19672459
Tomm40	0,39889	1,0848112	0,09116678
Tars2	0,280763	1,0523493	NA
Acadsb	0,599893	1,0262952	0,28414242
Fdx1	0,280763	1,0248786	NA
Sirt4	0,312715	0,9988482	NA
D2hgdh	0,495782	0,978324	NA
Ndufs4	0,293629	0,9781724	-0,15679461
Hspd1	0,330098	0,9400601	0,09673283
Mrpl45	0,320231	0,9347227	-0,07607517
Fabp3	0,315465	0,9346028	0,45051427
Atp5f1d	0,386904	0,905328	0,32742396
Ndufa12	0,51557	0,8268013	-0,34724204
Trak1	0,521078	0,7772172	0,24176825
Rab10	0,669586	0,7478115	0,54054898
Cox7a2	0,572851	0,7287986	1,48535044

Vars2	0,46779	0,7170921	0,18752902
Cat	0,507477	0,7042931	0,06050866
Ctsd	0,483149	0,6851963	-0,71921424
Ldhd	0,651223	0,6034217	NA
Myh6	0,543886	0,6002338	0,65952931
Pccb	0,572119	0,5971594	0,46811597
Cycs	0,527987	0,5761049	NA
Ywhae	0,739618	0,5098574	0,04206988
Ighm	0,621741	0,4917554	NA
Myom2	0,713452	0,4904926	0,28203171
Bdh1	0,651223	0,4593037	-0,44704946
Mrpl13	0,625807	0,4443854	-0,10851629
Atp5c1	0,669586	0,3927405	5,00E-05
Mrps36	0,741422	0,3836084	NA
Fis1	0,783607	0,3317174	0,51324202
Park7	0,81759	0,3117905	0,00757396
Prelid2	0,741422	0,3055199	-0,16274532
Lamc1	0,758729	0,2844704	0,5287584
Hsp90b1	0,849105	0,223191	NA
Shmt2	0,857363	0,1840997	0,75436682
Cryab	0,837829	0,1808333	-0,04780059
Acot9	0,837829	0,1803713	-0,11838661
Tomm22	0,868433	0,1446585	-0,03067709
Hsdl1	0,938639	0,1058461	-0,10596952
Clpx	0,947706	0,0805865	-0,13803961
Cps1	0,947706	0,0719743	NA
Mavs	0,950311	0,0577702	1,78901757
Hsp90ab1	0,95949	0,0568117	0,65099374
Rab1b	0,973458	0,0297501	NA
Calu	0,958106	-0,04574	0,78978854
Qdpr	0,950311	-0,063209	1,28916063
Hspa5	0,849105	-0,195341	0,2538068

Atic	0,906106	-0,228271	0,582148
Lonp1	0,809575	-0,244226	0,59322458
Rap1b	0,807952	-0,269749	NA
Mccc2	0,740326	-0,334722	0,34839469
Ldhb	0,788729	-0,339047	-0,00510539
Cyct	0,669878	-0,370647	NA
Myl6	0,768844	-0,398296	0,89692218
Ide	0,758278	-0,398695	NA
Nom1	0,64276	-0,425926	NA
Rplp0	0,741422	-0,436152	0,1623961
Hk2	0,625807	-0,455845	1,06809997
Cltc	0,741422	-0,511063	0,1092956
Rpl31	0,757776	-0,520502	NA
Maoa	0,669586	-0,522841	NA
Cad	0,621741	-0,541099	NA
Vim	0,605651	-0,552159	NA
Mrpl53	0,568544	-0,555685	NA
P4hb	0,70246	-0,560872	0,20643326
Arg1	0,543886	-0,578855	0,88536123
Rab5c	0,666482	-0,587382	0,1605778
Rpl23	0,559825	-0,63848	0,68798409
Rpl13	0,618003	-0,662365	NA
Ywhah	0,546491	-0,670496	0,37843263
Arf3	0,512525	-0,698098	0,21611898
Ociad1	0,456425	-0,753924	-0,13430152
Rpl10	0,568544	-0,817489	0,18956423
Rpl11	0,524636	-0,820118	NA
Nme2	0,507477	-0,843389	0,23322439
Rpl3	0,479182	-0,877657	-0,03571881
Idh1	0,349619	-0,899532	0,48332894
Rack1	0,470727	-0,914929	-0,00084766
Krt5	0,356878	-0,928035	0,71336642
Rps9	0,582765	-0,968437	NA

Rps3	0,46779	-0,972133	0,25167775
Sco1	0,434748	-0,998335	-0,11906997
Anxa6	0,543886	-0,998921	0,34358554
Gpd1	0,337105	-1,056118	0,3297055
Anxa2	0,285807	-1,099335	0,48278248
Rps8	0,427363	-1,110656	0,52016359
Rab7a	0,301549	-1,132676	0,01378458
Actb	0,284354	-1,155985	0,22653392
Rps16	0,391848	-1,16498	0,6083409
Hspe1	0,293808	-1,177697	0,39147887
Aldoa	0,339296	-1,19661	0,54190472
Ilf3	0,426508	-1,219273	1,11843993
Hspa1a	0,260232	-1,243412	0,61265345
Hnrnpk	0,25663	-1,249187	1,51277185
Prdx2	0,204253	-1,278542	-0,18003118
Mthfd1	0,425021	-1,279054	-0,30197773
Prdx6	0,369916	-1,283611	-0,25639062
Ssr4	0,315335	-1,284315	NA
Ddx3x	0,211487	-1,337232	-0,56753031
Myh9	0,315764	-1,376728	1,03921503
Rpl6	0,364051	-1,387759	1,0084159
Impa2	0,226315	-1,432246	0,69497908
Rps13	0,315764	-1,442536	0,64702276
Actn1	0,27735	-1,463038	1,33873402
Ppib	0,232388	-1,488362	0,37587076
Gapdh	0,2188	-1,572532	0,07458655
Pkm	0,184539	-1,607108	0,74248285
Dbi	0,142482	-1,641836	NA
Rplp2	0,120837	-1,659815	0,63175823
Uba1	0,309285	-1,761006	0,24042777
Hsp90aa1	0,204747	-1,785144	0,73855696
Tuba1a	0,238373	-1,802108	0,80797706
Rpl34	0,24184	-1,807517	0,04139266

Rpl34	0,24184	-1,807517	0,04139266
Ldha	0,146727	-1,81486	0,64038507
Fasn	0,148832	-1,867677	0,68425597
Eef2	0,203742	-1,913778	0,8329657
Rpl7	0,202523	-1,929082	0,0272542
Rpl18	0,169745	-1,99812	0,09079739
Ucp1	0,292054	-2,051982	1,83480565
Pdia3	0,099963	-2,097317	0,13519511
Rab11b	0,122126	-2,149091	NA
Txn1	0,059171	-2,157831	0,2367291
Calm3	0,040805	-2,289742	NA
Sod1	0,042717	-2,320084	NA
Anxa1	0,09283	-2,387749	-0,62050168
Pgk1	0,073798	-2,512086	0,09797089
Ywhaz	0,049843	-2,678315	-0,42179595
Eef1g	0,056845	-2,78065	0,59717076
Hspb1	0,055337	-2,822628	0,04329966
Tubb5	0,103418	-3,087091	1,45741391
Bak1	0,069571	-3,247829	NA
