# Supplementary material for: The intra-mitochondrial O-GlcNAcylation system rapidly modulates OXPHOS function and ROS release in the heart
Source: Commun Biol. 2022 Apr 12;5:349. doi: 10.1038/s42003-022-03282-3 (PMC9005719; doi:10.1038/s42003-022-03282-3)
Supplement: Supplementary file 2 — Description of Additional Supplementary Files [file 42003_2022_3282_MOESM2_ESM.pdf]

Description of Additional Supplementary Files

File name: Supplementary Data 1
Description: Source data for the graphs and charts.
