# Supplementary material for: The intra-mitochondrial O-GlcNAcylation system rapidly modulates OXPHOS function and ROS release in the heart
Source: Commun Biol. 2022 Apr 12;5:349. doi: 10.1038/s42003-022-03282-3 (PMC9005719; doi:10.1038/s42003-022-03282-3)
Supplement: Supplementary file 4 — Reporting Summary [file 42003_2022_3282_MOESM4_ESM.pdf]

nature portfolio

Corresponding author(s): Yan Burelle

Last updated by author(s): Feb 10, 2022

Reporting Summary

Nature Portfolio wishes to improve the reproducibility of the work that we publish. This form provides structure for consistency and transparency in reporting. For further information on Nature Portfolio policies, see our Editorial Policies and the Editorial Policy Checklist.

Statistics

For all statistical analyses, confirm that the following items are present in the figure legend, table legend, main text, or Methods section.

n/a	Confirmed	
☐	☒	The exact sample size (*n*) for each experimental group/condition, given as a discrete number and unit of measurement
☐	☒	A statement on whether measurements were taken from distinct samples or whether the same sample was measured repeatedly
☐	☒	The statistical test(s) used AND whether they are one- or two-sided<br>*Only common tests should be described solely by name; describe more complex techniques in the Methods section.*
☒	☐	A description of all covariates tested
☐	☒	A description of any assumptions or corrections, such as tests of normality and adjustment for multiple comparisons
☐	☒	A full description of the statistical parameters including central tendency (e.g. means) or other basic estimates (e.g. regression coefficient) AND variation (e.g. standard deviation) or associated estimates of uncertainty (e.g. confidence intervals)
☐	☒	For null hypothesis testing, the test statistic (e.g. *F*, *t*, *r*) with confidence intervals, effect sizes, degrees of freedom and *P* value noted<br>*Give P values as exact values whenever suitable.*
☐	☒	For Bayesian analysis, information on the choice of priors and Markov chain Monte Carlo settings
☒	☐	For hierarchical and complex designs, identification of the appropriate level for tests and full reporting of outcomes
☒	☐	Estimates of effect sizes (e.g. Cohen's *d*, Pearson's *r*), indicating how they were calculated

Our web collection on statistics for biologists contains articles on many of the points above.

Software and code

Policy information about availability of computer code

Data collection	Data from mass spectrometry were processed using Sequest HT search engine within Proteome Discoverer 2.4.
Data analysis	Proteomic analysis analysis was conducted in R (R Core Team, 2020). The differential expression between groups was statistically assessed through linear models using empirical Bayes methods for variance modelling, as implemented in the R/Bioconductor limma package.<br><br>Pathway analysis was performed using g:Profiler (Reimand 2007) platform. The g:SCS (shortest common superstring) algorithm was used for multiple hypothesis testing corrections using a default alpha threshold of 0.05. The ggplot2 R package was used to generate bubble plots in which pathway enrichments were expressed as Rich Factors.<br><br>Proteins interactions were determined from STRING (Doncheva 2019) and imported in The Cytoscape (version 3.8.0) stringApp plugin. Clustering of the STRING network was performed with the Markov Cluster (MCL) algorithm and pathways/processes were determined using the Auto-annotate plugin (Kucera 2016). Venn diagrams were generated using the Eulerr R Package.<br><br>Statistical analysis for the functionnal studies were performed using GraphPad Prism 8.4.3.

For manuscripts utilizing custom algorithms or software that are central to the research but not yet described in published literature, software must be made available to editors and reviewers. We strongly encourage code deposition in a community repository (e.g. GitHub). See the Nature Portfolio guidelines for submitting code & software for further information.

Data

Policy information about availability of data

All manuscripts must include a data availability statement. This statement should provide the following information, where applicable:

- Accession codes, unique identifiers, or web links for publicly available datasets
- A description of any restrictions on data availability
- For clinical datasets or third party data, please ensure that the statement adheres to our policy

The mass spectrometry proteomics data have been deposited to the ProteomeXchange Consortium via the PRIDE partner repository with the dataset identifier reviewer_pxd026495@ebi.ac.uk. Requests for access to other data should be addressed to senior authors: Yan Burelle (yburell2@uottawa.ca) and Luc Bertrand (luc.bertrand@uclouvain.be). All requests will need to specify how the data will be used and will require approval by co-investigators.

Field-specific reporting

Please select the one below that is the best fit for your research. If you are not sure, read the appropriate sections before making your selection.

☒ Life sciences ☐ Behavioural & social sciences ☐ Ecological, evolutionary & environmental sciences

For a reference copy of the document with all sections, see nature.com/documents/nr-reporting-summary-flat.pdf

Life sciences study design

All studies must disclose on these points even when the disclosure is negative.

Sample size	All experiments used a minimum of 3 separate experimental replicate (mice/mitochondrial isolation from rat) per group.
Data exclusions	For proteomic analysis , NButGT-treated replicate n°3 was removed as it did not respond to treatment (assessed by O-GlcNAc immunoblotting and exploratory analyses conducted in R).
Replication	Proteomic analysis was performed in a minimum of 3-4 distinct mice/rats per experimental group. For functional studies, minimum 3 rats per group were used.
Randomization	Animals were randomly allocated to experimental groups.
Blinding	Proteomics samples were coded and sent to the core facilities. The analysis was therefore blinded.

Reporting for specific materials, systems and methods

We require information from authors about some types of materials, experimental systems and methods used in many studies. Here, indicate whether each material, system or method listed is relevant to your study. If you are not sure if a list item applies to your research, read the appropriate section before selecting a response.

Materials & experimental systems

n/a	Involved in the study	
☐	☒	Antibodies
☒	☐	Eukaryotic cell lines
☒	☐	Palaeontology and archaeology
☐	☒	Animals and other organisms
☒	☐	Human research participants
☒	☐	Clinical data
☒	☐	Dual use research of concern

Methods

n/a	Involved in the study	
☒	☐	ChIP-seq
☒	☐	Flow cytometry
☒	☐	MRI-based neuroimaging

Antibodies

Antibodies used	OGT (Cell Signaling #5368), OGA (Santa Cruz Biotechnology #sc-135093), Alpha tubuline (Thermo Fisher #62204), TOM20 (Thermo Fisher #H00009804-MO1), Histone 3 (Cell Signaling #3638S), OXPHOS (Abcam #110413), NDUFS1 (#Proteintech #12444-1-AP) and O-GlcNAc-HRP (Abcam #201995).
Validation	NA

Animals and other organisms

Policy information about studies involving animals; ARRIVE guidelines recommended for reporting animal research

Laboratory animals	Twelve week-old mice (C57BL/6 N, male) from Janvier Labs.<br>Eight week-old rats (Wistar, male).
Wild animals	NA
Field-collected samples	NA
Ethics oversight	Experiments on rats were approved by the University of Ottawa Institutional Animal Care Committee and conducted according to the directives of the Canadian Council on Animal Care. Experiments on mice were approved by the Animal Research Committee of the Université catholique de Louvain and conformed to the American Heart Association Guidelines for Use of Animal in Research.

Note that full information on the approval of the study protocol must also be provided in the manuscript.
